# HIV causes global B cell dysregulation and restricts HBV-specific B cell development in an incident HBV cohort

**DOI:** 10.1172/JCI203138

**Published:** 2026-04-07

**Authors:** Katherine E. Cascino, Thomas Liechti, Eric C. Seaberg, Kathleen Stevens, Steven M. Wolinsky, Mallory D. Witt, Robbie B. Mailliard, Mario Roederer, Justin R. Bailey, Chloe L. Thio, Andrea L. Cox

**Affiliations:** 1 Department of Medicine, Johns Hopkins University School of Medicine, Baltimore, Maryland, USA.; 2 ImmunoTechnology Section, Vaccine Research Center, National Institute of Allergy and Infectious Diseases (NIAID), NIH, Bethesda, Maryland, USA.; 3 Department of Epidemiology, Johns Hopkins University, Baltimore, Maryland, USA.; 4 Division of Infectious Diseases, Department of Medicine, Feinberg School of Medicine, Northwestern University, Chicago, Illinois, USA.; 5 Lundquist Research Institute at Harbor-UCLA Medical Center, Torrance, California, USA.; 6 Department of Medicine, University of Pittsburgh School of Medicine, Pittsburgh, Pennsylvania, USA.

**Keywords:** Immunology, Virology, B cells

## Abstract

**BACKGROUND:**

Functional B cell responses for both prevention and control of hepatitis B virus (HBV) infection remain poorly understood, including in the context of HBV/HIV coinfection.

**METHODS:**

Here, we employed high-dimensional single-cell analysis to assess global and hepatitis B surface antigen–specific (HBsAg-specific) B cells in a longitudinal cohort of incident HBV from the Multicenter AIDS Cohort Study, with a subset of the cohort living with HIV-1.

**RESULTS:**

We observed that prior HIV infection has negative consequences for B cell function in early post-acute HBV infection, including increased frequencies of atypical memory B cells and regulatory B cells, expression of the activation marker CD86 on multiple B cell subsets in chronic HBV (CHB), and restricted expansion of HBsAg-specific B cells. In contrast, in HBV monoinfection, we observed no changes in the global B cell population from prior to infection and robust expansion of HBsAg-specific B cells. These expanded antigen-specific B cells resembled class-switched intermediate and resting memory B cells, with activation phenotypes that may contribute to ongoing HBV control.

**CONCLUSION:**

HIV infection has a significant impact on B cell responses to subsequent HBV infection that may promote development of CHB in HBV/HIV coinfection.

**FUNDING:**

Vaccine Research Center, NIAID, Bill & Melinda Gates Foundation, and NIH.

## Abstract 2

HIV infection has a significant negative impact on B cell responses to subsequent hepatitis B virus infection that may promote development of chronic HBV in co-infection.

## Introduction

After infection with hepatitis B virus (HBV), 95% of immunocompetent adults control the infection and develop protective antibodies, whereas the remaining 5% develop chronic HBV (CHB), a leading cause of liver disease and hepatocellular carcinoma (HCC) globally. B lymphocytes play a crucial role in the prevention and control of HBV infection through diverse effector functions, including the secretion of antibodies and cytokines (1). Antibodies against the HBV surface antigen (anti-HBsAg) indicate control of an acute infection and protect against infection when generated through vaccination (2, 3). Further, B cell–depleting immunotherapy can lead to HBV reactivation even years after recovery from an acute infection, highlighting the importance of B cells in maintaining control (4–6). Thus, B cells are considered a target for generating a functional cure. However, there is a limited understanding of what constitutes an effective B cell response to HBV.

There is some evidence of perturbations in the global B cell population in CHB, including increased proportions of activated CD69^+^CD73^+^ B cells (7, 8) and atypical memory (AtM) B cells, a CD21^–^CD27^–^ B cell population often associated with chronic antigenic stimulation (9, 10). Expansion of these populations has negative consequences, including hyperactivation and functional impairment thought to contribute to poor viral clearance. Furthermore, B regulatory cells (B_regs_) emerge in CHB and are associated with increased HBV replication (11, 12). However, insights into the functional role of B cells in an effective immune response to acute hepatitis B may be better gleaned from examining B cells early (prior to CHB development) and those specific for HBsAg. Peripheral HBsAg-specific B cells are present at similar proportions in acute, chronic, and controlled infection; on HBV treatment; and in vaccinated controls (9, 10, 13–15). However, in people with CHB, HBsAg-specific B cells have decreased proliferative capacity and higher proportion of AtM (mean 30% of HBsAg-specific population), with increased levels of inhibitory markers, including PD-1, BTLA, CD22, and FcRL5 (9, 10, 14, 15). While previous studies began to define phenotypes of HBsAg-specific B cells in CHB, it is not known if these phenotypic changes result from chronic infection or are causally associated with failure to recover during the acute infection.

Understanding the role of B cells in HBV recovery is also imperative in people who are living with HIV-1 due to the high prevalence of HBV/HIV coinfection and the adverse consequences of HIV on CHB (16). Because people with HIV are less likely to develop anti-HBsAg and recover from an acute infection (16, 17) and are more likely to undergo HBV reactivation (18, 19), understanding how HIV affects the HBV-specific B cell response may offer insights to an effective B cell response. Whether HBV-specific B cells are present and affected in HBV/HIV coinfection similarly to the global B cell compartment in HIV monoinfection, such as demonstrating increased proportions of activated subsets (plasmablasts, CD21^–^CD27^+^ activated memory [AM], AtM) (20) and inflammatory/adhesion molecules (CXCR3, CD11c) (20, 21) on these subsets, remains to be seen.

Because acute HBV infection is frequently asymptomatic, most reports on immune responses in HBV have been confined to assessment of CHB samples isolated years after acute HBV infection. This raises the question of whether immune signatures observed in these cohorts are a *cause* of failed HBV control or a *consequence* of prolonged HBV exposure. To address this knowledge gap, we assessed a unique longitudinal cohort of participants living with or without HIV who acquired an incident HBV infection. We developed a high-dimensional flow cytometry panel to assess global and HBsAg-specific B cells in these participants (22). We interrogated B cell dynamics at the single-cell and antigen-specific levels to improve our understanding of B cell characteristics and the role of B cell immunity around the acute HBV infection period, including in the context of HBV/HIV coinfection.

## Results

### Demographic and clinical characteristics.

To investigate B cell characteristics associated with HBV outcome, we studied cryopreserved peripheral blood mononuclear cells (PBMCs) from 76 men (44 with HIV-1 and 32 without HIV-1) with incident HBV infection in the Multicenter AIDS Cohort Study (MACS), now the MACS-WIHS Combined Cohort Study (MWCCS) (23, 24). MACS participants were enrolled if they were at risk of or living with HIV-1 and were prospectively followed at semiannual study visits (see Methods). As previously reported for the overall MACS cohort, a subset of men acquired HBV during follow-up (25). Of men with incident HBV infection, a higher proportion of men with HIV (MWH) not on active HBV-active antiretroviral therapy (ART) developed CHB (17.5%; 95% CI 8.7%–29.9%) than men without HIV (MWoH) (8.2%; 95% CI 3.8–15.0%) (26). For this study, sampling procedures did not randomly select MACS participants and, therefore, do not reflect similar HBV recovery probabilities (see Methods). Rather, given the limited number of participants who progressed to CHB, individuals who met the sampling requirements were *all* selected for assessment in this study, followed by matching of controllers (see Methods). Of the 44 MWH, 31 controlled the HBV infection (70%) and 13 progressed to CHB (30%). Of the 32 MWoH, 23 controlled the HBV infection (72%) and 9 progressed to CHB (28%). Demographic characteristics stratified by HIV-1 status are in Table 1.

**Figure 1:**
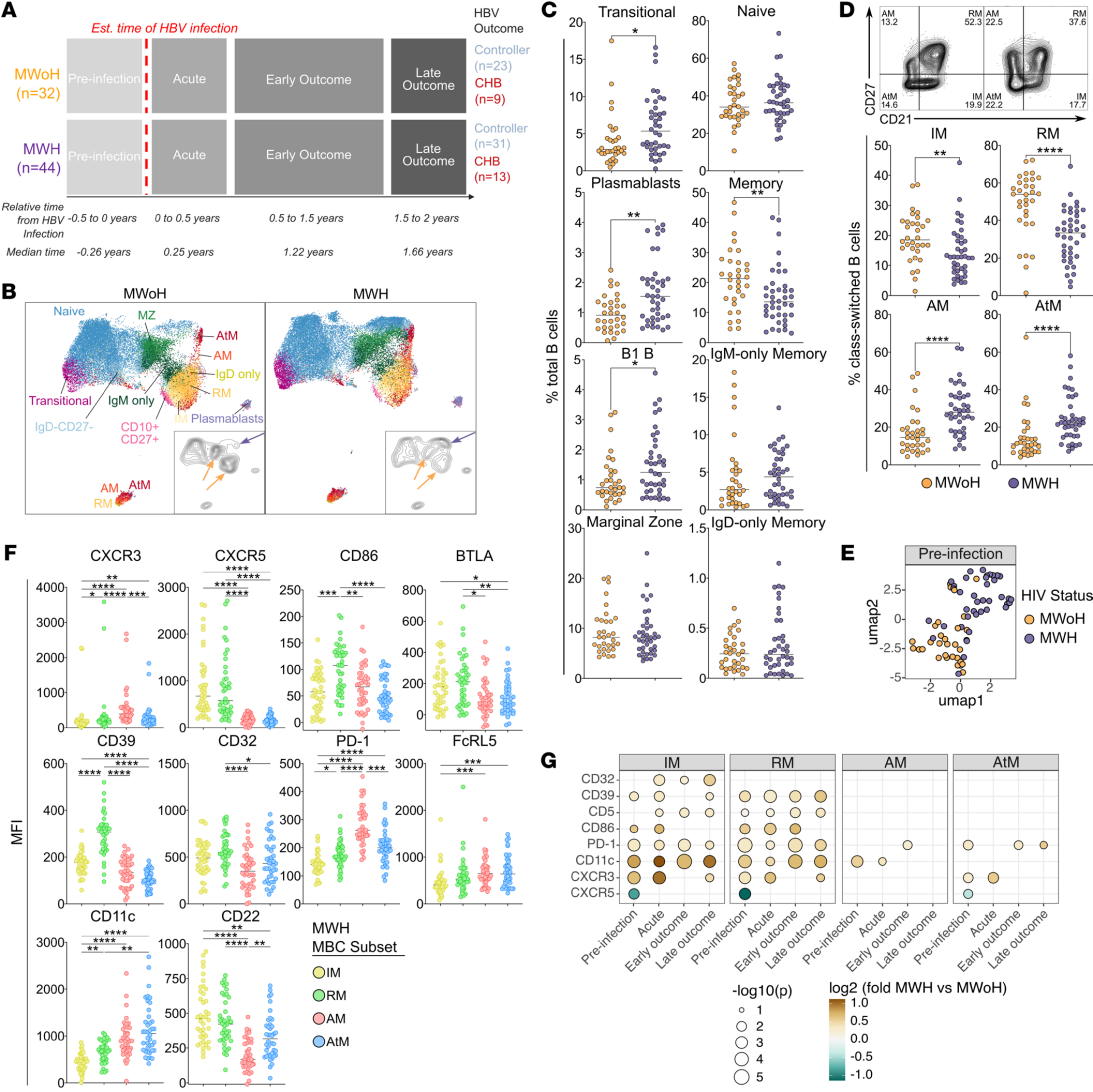
Global B cell subsets are significantly altered in MWH. (**A**) Schematic overview of longitudinal HBV cohort. (**B**) Concatenated flow cytometry data depicted as UMAP of all B cells from preinfection samples and plotted for MWoH or MWH. Each dot represents a single B cell. All markers except the dump/viability and HBsAg probes were used to compute UMAP. Contour plots depict distribution of events on UMAP based on the equally sampled preinfection data. Yellow and purple arrows indicate areas of up- and downregulation in MWoH and MWH, respectively. (**C**) Frequencies of B cell subsets from MWoH (orange) and MWH (purple). (**D**) Representative MWoH participant (left plot) and MWH (right plot) from the preinfection time point were examined for expression of MBC subset markers CD27 and CD21. Frequencies of MBC subsets from all MWoH and MWH are shown below. (**E**) UMAP based on the composition of all B cells shown in **C** and **D**. Each dot represents 1 individual at preinfection time point. (**F**) Mean fluorescence intensity (MFI) of all phenotypic markers on MBC subsets based on CD21/CD27 expression profile from MWH pre-HBV infection. (**G**) Bubble plot highlights *P* values of all significant phenotypic marker expression (MFI) on MBCs between MWoH and MWH when compared using Wilcoxon’s signed-rank test. Dot size is based on –log10-transformed and unadjusted *P* values, and dot color is based on log2-transformed (upregulated in MWH, brown; MWoH, blue) fold-change. (**B**–**F**) Data from preinfection samples for MWoH (*n* = 32) and MWH (*n* = 44). (**C** and **D**) Data compared using Mann-Whitney *U* test and (**F**) Kruskal-Wallis test corrected for multiple comparisons, where each dot represents a single measurement from each individual assessed at the preinfection time point. Bars represent median values. *, *P* < 0.05; **, *P* < 0.01; ***, *P* < 0.001; ****, *P* < 0.0001.

For each participant, up to 4 time points were analyzed, including (i) pre-HBV infection, (ii) acute HBV infection, (iii) early outcome, and (iv) late outcome, with the latest outcome time points acquired less than 2 years from the estimated time of incident HBV infection (Figure 1A). At each time point, we utilized a 24-color flow cytometry assay to define main B cell subsets, including global unswitched and class-switched memory and HBsAg-specific B cells (Supplemental Figure 1; supplemental material available online with this article; https://doi.org/10.1172/JCI203138DS1) (22).

For both MWoH and MWH, the acute time point consisted of 3 groups based on serologic analysis and eventual outcome of HBV infection (controller 1, controller 2, and CHB). Participants in the controller 1 and CHB groups were HBsAg positive with or without positive HBV DNA while the controller 2 group was already HBsAg negative and anti–hepatitis B core (anti-HBc) positive. Thus, the controller 2 group both acquired and controlled their incident HBV infection (as shown by HBsAg clearance) in the 6 months between preinfection and acute sampling (Supplemental Figure 2A and Table 2). There was no difference in time between the preinfection visit and the acute visit in any of the 3 acute groups (controller 1: median = 0.50 years, IQR = 0.48–0.56 years; controller 2: median = 0.52 years, IQR = 0.50–0.62 years; chronic: median = 0.51 years, IQR = 0.48–0.57 years; *P* value = 0.25). Due to lack of significant differences in expression of B cell characteristics despite their differential serological status between controller 1 and controller 2 (Supplemental Figure 2, B–D), we combined the 2 controller groups for subsequent analysis.

### Global B cells are dysregulated in HIV-1 infection.

We first determined HIV-1–dependent B cell perturbations before HBV infection in MWH by comparing them with MWoH. Uniform manifold approximation and projection–based (UMAP-based) dimensionality reduction revealed differences in the B cell compartment as demonstrated by different increased density areas between MWoH and MWH (Figure 1B). MFI heatmap overlays for all panel markers are shown (Supplemental Figure 3A). B cell annotation based on manual gating revealed that areas of increased density among MWoH were represented by both unswitched B cell populations (IgM^+^IgD^+^CD27^+^ marginal zone [MZ] and IgM^+^IgD^–^CD27^+^ IgM-only memory) and class-switched populations (CD21^+^CD27^–^ intermediate memory [IM] and CD21^+^CD27^+^ resting memory [RM]), while the area of increased density among MWH consisted of IgD^+^CD27^–^ naive (unswitched), AM, and AtM (class-switched) subsets. We next confirmed these dynamics by quantifying the frequencies of all traditional B cell subsets pre-HBV infection for total B cells (Figure 1C) and class-switched B cells (Figure 1D). MWH showed increased frequencies of transitional B cells (CD10^+^CD27^–^), B1 B cells (CD24^–/+^CD38^–/int^CD27^+^CD43^+^), and plasmablasts (CD24^–^CD38^+/++^) and decreased levels of total memory B cells (MBC) compared with MWoH (Figure 1C). The class-switched MBC compartment for MWH was skewed toward activation, indicated by fewer IM and RM B cells and increased frequencies of AM and AtM B cells compared with MWoH (Figure 1D). UMAP analysis based on these differential B cell subset abundances resulted in clear separation of MWH and MWoH (Figure 1E). This segregation based on HIV-1 status persisted after HBV infection in the acute and early/late outcome stages, supporting that HBV infection does not change all global B cell differences between MWH and MWoH (Supplemental Figure 3B).

We further investigated the expression of functional markers on MBC subsets in MWH pre-HBV infection (Figure 1F and Supplemental Figure 3C). These markers included chemokine receptors (CXCR3, CXCR5), the costimulatory molecule CD86, regulatory molecules (BTLA, CD39), inhibitory Fc receptor CD32, and inhibitory markers associated with chronic infections (PD-1, FcRL5, CD11c, CD22) (22). Consistent with the HIV-1 literature (20, 21), in MWH, AM B cells expressed more CXCR3 and PD-1 than AtM, while both AM and AtM expressed higher levels of FcRL5 and CD11c compared with IM and RM (Figure 1F). These phenotypic expression patterns are also observed on AtM B cells in other chronic infections (21). In contrast, CXCR5, CD86, BTLA, CD39, CD32, and CD22 showed higher expression on IM and/or RM B cells compared with AM and AtM (Figure 1F). We also examined expression patterns on MBC subsets of MWoH before HBV infection (Supplemental Figure 3, D and E) and compared with MWH (Figure 1G). The expression of activation/exhaustion markers (e.g., CD86, PD-1, CD11c) on IM and RM B cells in MWH were increased compared with MWoH irrespective of HBV infection stage (Figure 1G). Overall, the pre-HBV infection samples demonstrate a B cell compartment skewed toward activation or exhaustion in MWH, consistent with previous reports. Most of the phenotypic B cell differences between men with and without HIV were in the IM and RM subsets and persisted throughout the HBV infection course.

### Limited global B cell characteristics correlate with HBV outcome.

**Figure 2:**
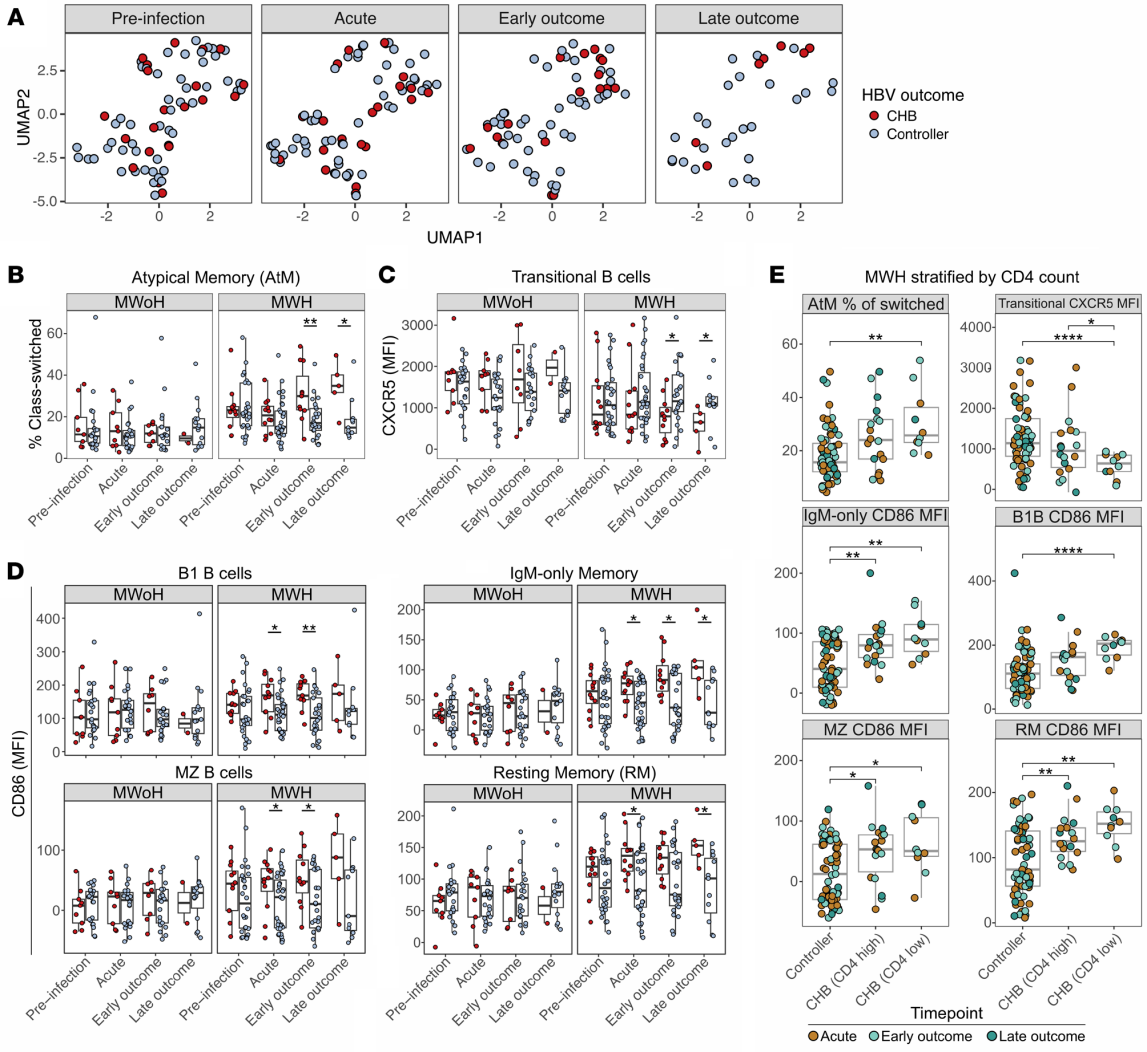
Differences by HBV outcome in global B cell populations are minimal and observed only in HIV-1 coinfection. (**A**) UMAP of B cells shown in Figure 1, C and D, from all MWoH and MWH at each time point (CHB, red; controller, blue). At each time point, each participant is represented by 1 dot. (**B**–**D**) Abundance of AtM (% class-switched) and expression (MFI) of CXCR5 (transitional B cells) and CD86 (B1, IgM-only memory, MZ, and RM B cells) between HBV outcome in MWH and MWoH are shown. At each time point, each participant is represented by 1 dot (MWoH: preinfection, controller *n* = 23, CHB *n* = 9; acute, controller *n* = 22, CHB *n* = 9; early outcome, controller *n* = 21; CHB *n* = 8; late outcome, controller *n* = 15, CHB *n* = 2; MWH: preinfection, controller *n* = 28, CHB *n* = 12; acute, controller *n* = 31, CHB *n* = 12; early outcome, controller *n* = 25; CHB *n* = 11; late outcome, controller *n* = 11, CHB *n* = 5). Box plots show median, quartile, and minimum/maximum. Data compared using Wilcoxon’s signed-rank test. *P* values are unadjusted for multiple comparisons. (**E**) Manually gated B cell characteristics compared between MWH controllers and MWH CHB with low (<350 cells/mL) and high (>350 cells/mL) CD4^+^ counts using a generalized estimating equation (GEE) regression model. Each dot represents 1 sample, colored by time point (orange, acute; light green, early outcome; dark green, late outcome). *P* values were obtained from the GEE model. *, *P* < 0.05; **, *P* < 0.01; ****, *P* < 0.0001.

Having established baseline differences in global B cells based on HIV-1 infection, we next sought to interrogate global B cell dynamics throughout the course of HBV infection to identify features associated with HBV outcome. UMAP based on the abundance of all manually gated B cell subsets across each time point, irrespective of HIV-1 status, revealed considerable overlap by HBV outcome alone between controllers and CHB (Figure 2A). Thus, overall B cell subset composition was not associated with control of an HBV infection. Given the observed effect of HIV-1 on the B cell compartment, we performed all subsequent analyses comparing HBV outcome by first stratifying on HIV-1 status.

To gain more granular insights into HBV-dependent immune dynamics, we compared all manually defined immune characteristics (frequency and MFI; *N* = 370) at each time point between controllers and CHB, stratified by HIV-1 (see Methods and Supplemental Table 1). Statistically significant differences were observed most often during the post-acute period when comparing CHB with controllers, and nearly all were in MWH. Of the characteristics identified, the frequency of AtM B cells and CXCR5 expression on transitional B cells were significantly higher and lower, respectively, at outcome time points in those with CHB compared with controllers (Figure 2, B and C). In addition, expression of CD86 on multiple unswitched and memory B cell populations was significantly higher in MWH who developed CHB compared with controllers at both acute and outcome time points (Figure 2D and Supplemental Figure 2, C and D). Our results show that the differential immune signature signified by increased CD86 expression occurs during acute HBV infection between individuals who develop CHB versus control and persists in the post-acute period. Additional signatures including increased frequency of AtM B cells and decreased expression of CXCR5 on transitional B cells are observed only in the post-acute period.

For each of the characteristics identified above in MWH, we next stratified participants progressing to CHB based on CD4^+^ count and observed that those with low CD4^+^ counts (<350 cells/mL) had significantly higher frequencies of AtM B cells and CD86 expression or lower CXCR5 expression compared with controllers (Figure 2E). CXCR5 expression on transitional B cells was also significantly lower in CD4-low CHB participants when directly compared with CD4-high (>350 cells/mL) CHB participants. Taken together, MWH who progressed to CHB had higher frequencies of AtM B cells and CD86 expression on multiple B cell subsets and lower CXCR5 expression on transitional B cells at acute or outcome time points. These observations were significantly more pronounced with lower CD4^+^ count (Figure 2E). In contrast, MWoH had stable proportions of cells and expression of these molecules throughout their HBV infection regardless of HBV outcome.

### Unsupervised clustering reveals a unique population of B_regs_ upregulated in CHB at outcome in MWH.

Manual gating analysis is limited to biological knowledge and does not take the full dimensionality of the data into account (27). Thus, interesting immune phenotypes may be missed. To capture the phenotypic heterogeneity of B cells, we used unsupervised clustering (FlowSOM) from B cells in all 244 samples (17.5 million cells) to define 40 unique clusters (28). Overlay of the cluster annotation on UMAP of global B cells from all time points revealed heterogenous B cell phenotypes based on their unique locations on the UMAP (Supplemental Figure 4A). The average FlowSOM cluster frequency ranged from <0.1% to >30%, and a heatmap of all markers for each cluster is shown (Supplemental Figure 4B).

**Figure 3:**
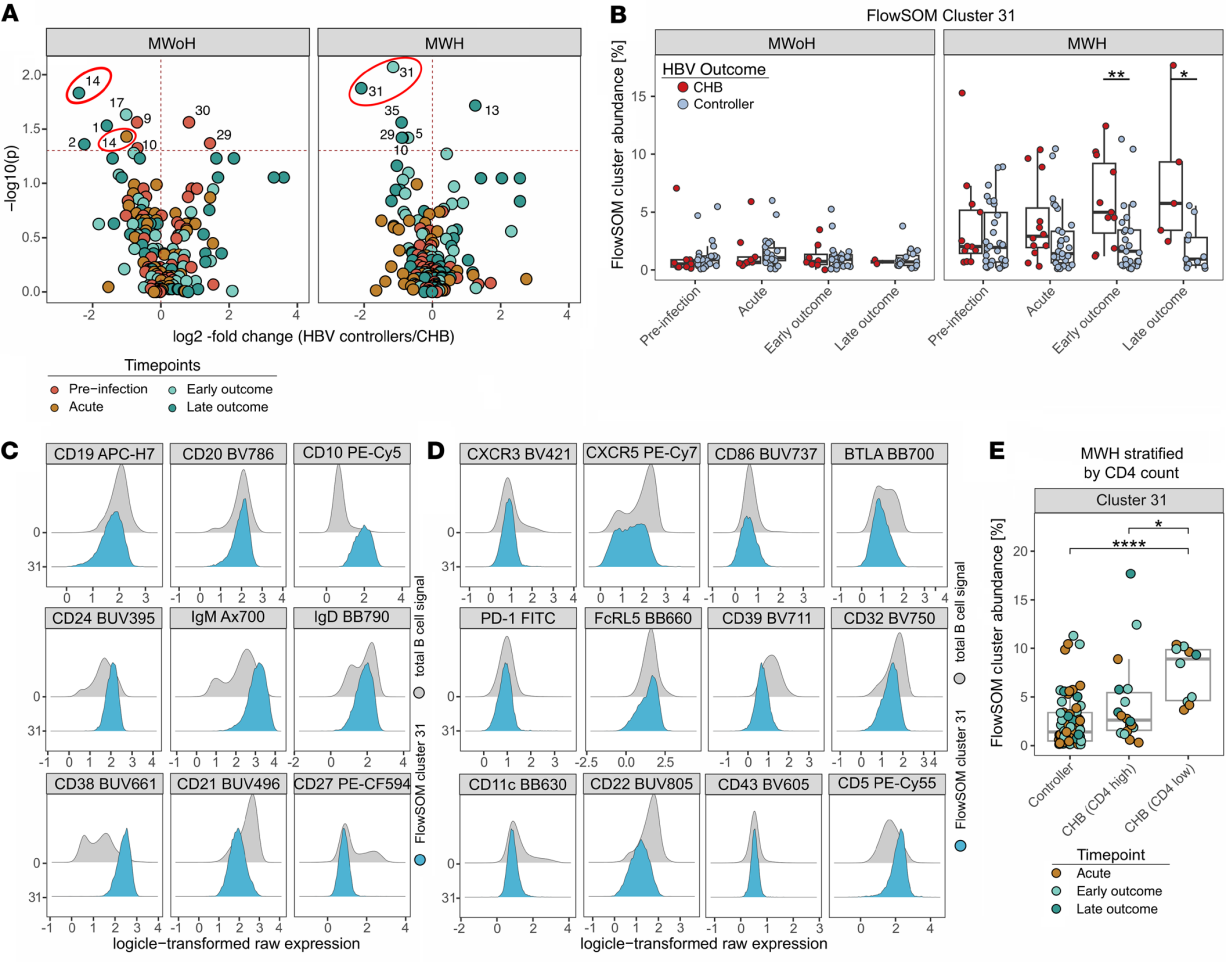
Unsupervised FlowSOM clustering reveals a B_reg_ population upregulated in MWH who develop CHB. (**A**) Comparison of FlowSOM cluster abundance by HBV outcome in MWoH and MWH by Wilcoxon’s signed-rank test is shown. Volcano plot depicts –log10-transformed unadjusted *P* values and log2 fold-change of cluster abundance in controllers compared with CHB. Upper right quadrant depicts clusters significantly upregulated in controllers while upper left quadrant depicts clusters significantly upregulated in CHB. Each dot represents a FlowSOM cluster colored by time point (red, preinfection; orange, acute; light green, early outcome; dark green, late outcome). (**B**) FlowSOM cluster 31 abundance in MWoH and MWH stratified by HBV outcome is shown. Each dot represents 1 sample for each participant at given time point (see Figure 2 legend for sample numbers). Box plots show median, quartile, and minimum/maximum. Data compared using Wilcoxon’s signed-rank test. *P* values are unadjusted for multiple comparisons. (**C** and **D**) Histogram plots show expression of all lineage markers (**C**) and phenotypic markers (**D**) for total B cells (gray) compared with FlowSOM cluster 31 (blue). (**E**) FlowSOM cluster 31 abundance compared between MWH controllers, MWH CHB with high CD4^+^ counts (>350 cells/mL), and MWH CHB with low CD4^+^ counts (<350 cells/mL) using a GEE regression model. Each dot represents 1 sample, colored by time point. *P* values were obtained from the GEE model. *, *P* < 0.05; **, *P* < 0.01; ****, *P* < 0.0001.

Next, we assessed if FlowSOM cluster abundance differed based on HBV outcome at any time point. We again delineated individuals based on HIV-1 status due to profound HIV-dependent B cell perturbations. Multiple clusters significantly differed between HBV controllers and CHB groups in either MWH or MWoH (*N* = 12) (Figure 3A). Marker expression for these clusters is shown (Supplemental Figure 4C), and the heatmap revealed further clustering into 3 groups (Supplemental Figure 4D), driven by CD11c, CD27, and CD10, respectively, as shown by principal component analysis (Supplemental Figure 4E).

While multiple clusters were significantly altered at a given time point, only 2 clusters, 14 and 31, differed at multiple time points and were selected to further explore (see Methods). Clusters 14 and 31 were significantly increased in CHB compared with controllers at multiple time points for MWoH and MWH, respectively (Figure 3A, red circles). Cluster 14 differed at nonconsecutive acute and late outcome in MWoH and was characterized by IgM^+^, IgD^+^, CD27^lo^, CD21^lo^, CXCR5^+^, CD11c^+^, and FcRL5^+^, likely representing activated naive B cells (Supplemental Figure 4C) (29). In contrast, cluster 31 differed consecutively at both early and late outcome time points in MWH, supporting a robust association with CHB (Figure 3B). Cluster 31 consisted of transitional-like B cells (CD10^+^IgM^+^IgD^+^CD5^hi^CD24^hi^CD38^hi^CD21^–^CD32^–^CD27^–^) with heterogenous expression of functional markers (Figure 3, C and D). Among MWH, cluster 31 abundance was also significantly higher in those with low CD4^+^ count progressing to CHB compared with either those with high CD4^+^ count progressing to CHB or those in the controller group (Figure 3E). This combination of lineage markers in association with progression to CHB suggests that cluster 31 represents B_regs_. Although production of IL-10 is the gold standard to identify B_regs_, transitional B cells expressing CD24, CD38, and CD5, as the cluster 31 cells do, have been previously shown to secrete IL-10 and have been described as B_reg_ populations (30). Together, FlowSOM cluster analysis reveals a likely unique B_reg_ population in MWH with significantly higher abundance in CHB versus controllers and in MWH with low versus high CD4^+^ counts.

### HBsAg-specific B cells expand in MWoH who control HBV and comprise distinct clusters.

**Figure 4:**
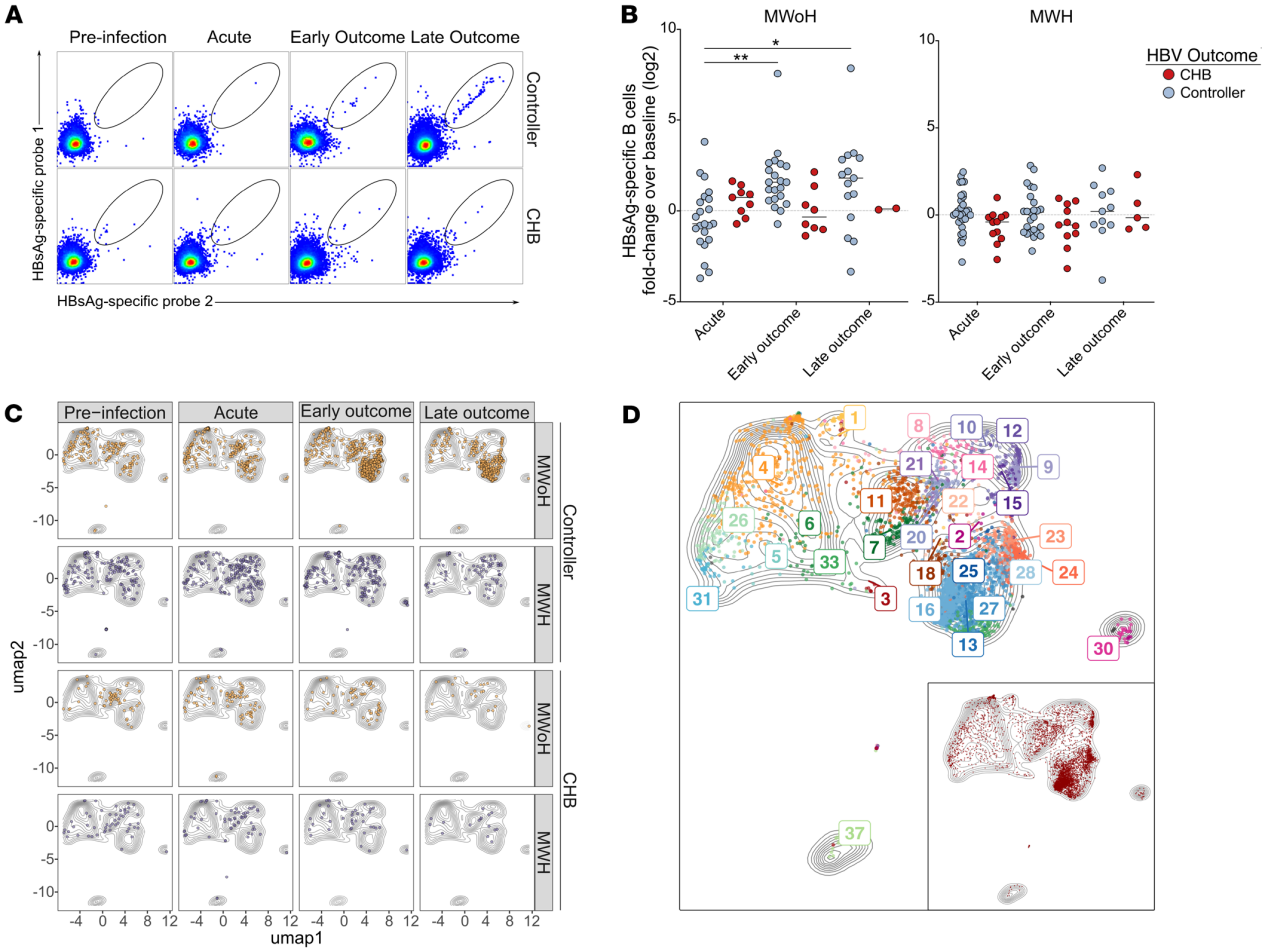
HBsAg-specific B cells are expanded in MWoH upon control of acute HBV infection. (**A**) Flow cytometry plots of manually gated HBsAg-specific B cells from 2 representative participants are depicted (MWoH controller, top row; MWoH CHB, bottom row). Dual-staining strategy with HBsAg probes was used to define antigen-specific B cells. (**B**) Frequencies of HBsAg-specific B cells plotted as log2 fold-change over baseline. Data compared using Kruskal-Wallis test corrected for multiple comparisons. Each dot represents 1 participant’s sample compared with baseline for each given time point (see Figure 2 legend for sample numbers). Bars represent median values. *, *P* < 0.05; **, *P* < 0.01. (**C**) UMAP of total B cells (contour plot, gray) overlaid with HBsAg-specific B cells (MWoH, yellow; MWH, purple) stratified by time point in controllers (top 2 rows) and CHB (bottom 2 rows). (**D**) UMAP of total B cells (contour plot, gray) overlaid with total HBsAg-specific B cells colored by FlowSOM cluster. Total HBsAg-specific B cells depicted in red on UMAP inlay. (**C** and **D**) Each dot represents a single HBsAg-specific B cell.

We next interrogated HBsAg-specific B cell dynamics and phenotypes based on HBV outcome given their importance in prevention and control of HBV. Representative manual gating of HBsAg-specific B cells using a dual-probe strategy is shown (Figure 4A). HBsAg-specific B cells ranged from undetectable to 1.13% total B cells (median 0.013%). Longitudinal sampling enabled us to uniquely assess the HBsAg-specific B cell dynamics within each participant by calculating the fold-change of frequencies over baseline (pre-HBV infection) (Figure 4B). We observed significant expansion in HBsAg-specific B cells in MWoH only in participants who controlled their HBV infection. No expansion was observed in MWoH who progressed to CHB, or in MWH, regardless of HBV outcome.

We also examined HBsAg-specific B cell frequencies cross-sectionally, similar to previously published analyses (9, 10, 13, 14). Here, we observed that MWoH who controlled their HBV infection showed significantly higher HBsAg-specific B cell frequencies at outcome time points compared with pre- and acute infection while CHB did not show an increased abundance of HBsAg-specific B cells (Supplemental Figure 5A). In contrast, MWH who ultimately controlled HBV infection had significantly higher HBsAg-specific B cell frequencies compared with CHB only during acute infection, not at outcome time points. When comparing MWoH and MWH, we observed significantly increased HBsAg-specific B cell frequencies in MWoH during the acute stage in those who progressed to CHB and during early outcome in the HBV controllers (Supplemental Figure 5B). HBsAg-specific B cell frequencies were not altered in MWH on treatment (*N* = 8, Supplemental Figure 5C). Stratifying the 3 acute groups (controller 1, controller 2, and chronic) revealed a significantly higher HBsAg-specific B cell frequency in controller 2 compared with participants with subsequent chronic HBV infection within MWH (Supplemental Figure 5D).

We then stratified the previously generated UMAP of total B cells based on time point, HIV-1 status, and HBV outcome and overlaid HBsAg-specific B cells (Figure 4C). We observed that the HBsAg-specific B cell expansion in MWoH occurred in specific UMAP locations, suggesting unique B cell subsets. HBsAg-specific B cells were detected at various frequencies in 39 of the 40 FlowSOM clusters (Supplemental Figure 5E). UMAP and FlowSOM analysis revealed that expanded HBsAg-specific B cells in MWoH controllers consist of multiple phenotypically distinct B cell subsets, primarily RM and IM B cells (Figure 4D and Supplemental Figure 4B for FlowSOM cluster marker expression patterns).

Overall, unlike the global B cell analysis demonstrating HBV outcome–related differences primarily in MWH with CHB, HBsAg-specific B cells expanded only in MWoH controllers. These cells expanded within distinctive areas of the UMAP, indicating potentially protective phenotypes that merited further interrogation.

### Expanded HBsAg-specific B cells are phenotypically heterogeneous.

**Figure 5:**
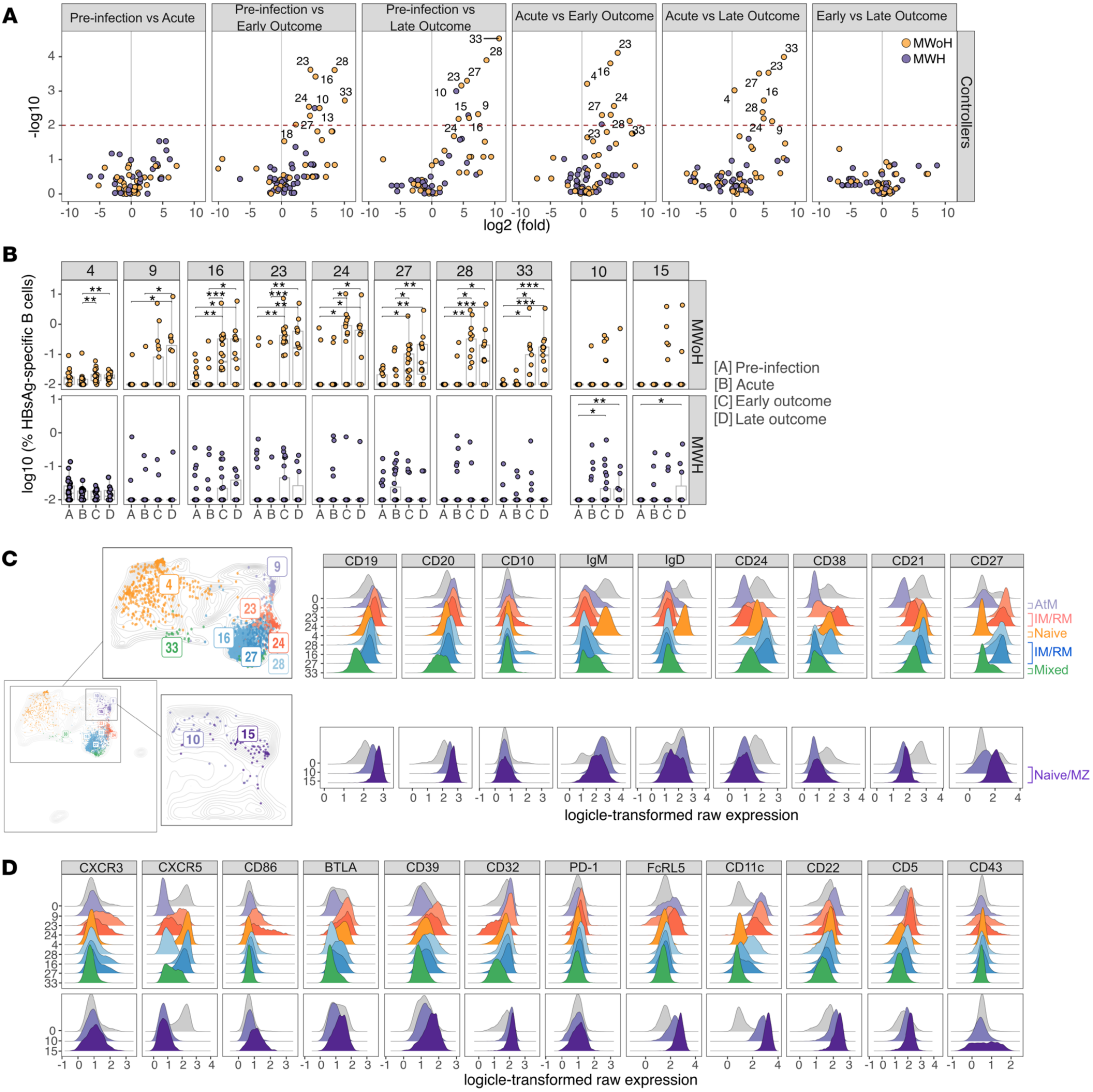
Expanded HBsAg-specific B cells are phenotypically heterogeneous. (**A**) Volcano plots depict log2 fold-change and –log10-transformed unadjusted *P* values based on comparisons of HBsAg-specific B cell abundance by FlowSOM cluster between each possible combination of time points in HBV controllers. *P* values calculated by Wilcoxon’s signed-rank test. Each dot represents a FlowSOM cluster, colored by HIV-1 infection status (MWoH, yellow; MWH, purple). (**B**) The log10-transformed frequency of HBsAg-specific B cells over time is plotted for both MWoH and MWH controllers in 10 clusters of interest. Top row consists of clusters upregulated in MWoH; bottom row are clusters upregulated in MWH. Abundances lower than 0.01% were adjusted to 0.01% for visualization but not statistical analysis purposes. Each dot represents 1 sample at a given time point. Data compared by Wilcoxon’s signed-rank test with Bonferroni’s correction for multiple testing. *, *P* < 0.05; **, *P* < 0.01; ***, *P* < 0.001. (**C**) Previously generated UMAP of total B cells overlaid with HBsAg-specific B cells from 10 clusters of interest are colored by FlowSOM cluster. HBsAg-specific B cells are plotted on zoomed-in sections of total UMAP (top row: expanded in MWoH; bottom row: expanded in MWH). (**C** and **D**) Histogram plots show expression levels for all lineage markers (**C**) and phenotypic markers (**D**) assessed in the panel for each of the 10 FlowSOM clusters compared with total B cells (gray histograms).

Next, we assessed if the frequency of HBsAg-specific B cells in any of the 39 FlowSOM clusters differed between time points to identify specific phenotypes associated with expansion upon HBV infection. Volcano plots stratified by HBV outcome and HIV-1 infection status revealed significant expansion of HBsAg-specific B cells in 12 FlowSOM clusters only in HBV controllers (Figure 5A). No differences occurred in CHB (Supplemental Figure 5F). All but 2 of the clusters identified were from MWoH and significantly differed between preinfection/acute versus outcome time points. This is consistent with our data showing expansion of total HBsAg-specific B cells in MWoH at the HBV infection outcome time points versus preinfection. Grouping the 10 clusters that significantly differed by at least 2 time points (see Methods) based on HIV-1 status (MWoH, clusters #4, #9, #16, #23, #24, #27, #28, #33; MWH, clusters #10 and #15) demonstrated significantly increased frequencies only during outcome time points (Figure 5B). The total number of B cells per sample was consistent across time points, regardless of HIV-1 status and HBV outcome, indicating that HBsAg-specific B cell frequencies did not merely reflect sampling differences (Supplemental Figure 5G).

We next sought to investigate the phenotypes of these clusters to gain in-depth insights into the phenotypic landscape of antigen-specific B cell expansion in HBV infection (Figure 5, C and D). Expression levels of all markers assessed in the panel were plotted by histogram and compared with total B cells (cluster 0, gray histogram). For MWoH, significantly expanded HBsAg-specific B cells consisted of clusters that resembled IgM^+^IgD^+^CD21^+^CD27^–^ naive B cells (cluster #4), IgM^–^IgD^–^CD21^+/int^CD27^+^ IM and RM B cells (clusters #16, #23, #24, #27, and #28), and IgM^–^IgD^–^CD21^–^CD27^lo^ AtM B cells (cluster #9) (Figure 5C). Cluster 33 appeared to be a mixed cluster consisting of negative to intermediate expression of all lineage markers assessed. For clusters upregulated in MWH (#10 and #15), both demonstrated lineage marker expression of IgM^+^IgD^int^CD21^–^CD27^int/+^ consistent with activated naive and/or MZ B cells.

Further analysis of phenotypic markers demonstrated heterogeneous phenotypes between the IM and RM B cell clusters from MWoH (Figure 5D). For example, clusters 23 and 24 showed higher expression of CD86, CD11c, and FcRL5, markers of both activation and exhaustion, compared with clusters 16, 27, and 28. In addition, clusters 16 and 27 are characterized by increased expression of lymph node trafficking marker CXCR5 compared with 23 and 24. In contrast, clusters 10 and 15, which resemble naive and/or MZ B cell subsets in MWH, demonstrated expression of multiple inhibitory and/or putative inhibitory markers, including CD11c, FcRL5, CD22, and CD32 (Figure 5D). Interestingly, no selected clusters expressed PD-1, and unlike the global B cell analysis, there was limited CD86 expression in these clusters.

Overall, in MWoH, HBsAg-specific B cell expansion was observed upon HBV outcome, and these cells demonstrate heterogeneous phenotypes associated mainly with resting and intermediate MBCs. For the 2 unswitched B cell clusters that minimally expanded in MWH, their phenotypic marker expression is consistent with dysfunctional naive-like HBsAg-specific B cell subsets. As these clusters were identified in controllers, the phenotypes may represent a suboptimal B cell compensatory mechanism in people with HIV-1 to manage subsequent infections.

## Discussion

Here, we provide a comprehensive longitudinal assessment of global and HBsAg-specific B cell dynamics in peripheral blood sampled before, during, and after established outcome for acute HBV infection. Our study evaluates B cell function in the largest longitudinal collection of participants with incident HBV. Importantly, over half of the participants included were living with HIV-1 at the time of HBV infection, allowing us to additionally interrogate B cell characteristics in the context of HBV/HIV coinfection. Prior studies have described widespread HIV-associated B cell perturbations (20, 31–33), which we confirmed in our cohort and followed through an acute HBV infection, demonstrating that HIV-1 negatively affects HBV infection responses, at both the global and the antigen-specific B cell level. Interestingly, no global B cell characteristics significantly differed in MWoH based on HBV outcome. This suggests that in monoinfection, global B cells remain minimally affected around the acute infection period, consistent with observations that people with CHB can respond normally to vaccination or subsequent infections (34, 35).

Multiple global B cell characteristics significantly differed based on HBV outcome between CHB and controllers in MWH. We identified decreased expression of CXCR5 on transitional B cells, increased frequencies of B_regs_, elevated CD86 expression on multiple unswitched and MBC subsets, and increased AtM B cell frequencies in individuals with CHB. These differences were not present prior to HBV infection, developing in MWH only after incident HBV, suggesting that baseline B cell differences did not predispose to CHB development and that precedent HIV-1 infection may drive adverse B cell changes over the course of CHB.

The lower expression of CXCR5 on transitional B cells observed in our study may impair replenishment of B cells. CXCR5 recruits mature B cells to lymph nodes to promote entrance into germinal centers for mounting productive antiviral responses (36). CD10^+^ transitional B cells are elevated in chronic viral infections, potentially as a compensatory reaction to the loss of naive B cells and to increased B cell activation (37). However, the role of CXCR5 on transitional B cells and its implications in HBV infection warrants further investigation.

In addition, we observed higher frequencies of B cells with phenotypes consistent with B_regs_ in CHB compared with controllers in MWH. While FlowSOM cluster 31 shows similarities with transitional B cells based on CD10, CD24, and CD38, their unique phenotypic characteristics suggest regulatory functions, as described previously. For instance, CD5 expression has been associated with reduced T cell proliferation and increased expression of IL-10 upon in vitro stimulation (38, 39). In addition, the reduced expression of CD21 and CD32 on cluster 31 matches the phenotypic characteristics of CD24^+^CD38^+^ transitional B cells with regulatory functions (40). Thus, based on these observations, we concluded that cluster 31 consists of B_regs_. B_regs_ have been reported in CHB monoinfection and contribute to a dampened CD8^+^ T cell response that may allow chronic infection to persist (11, 12). The CD8^+^ T cell response is crucial for control of HBV, and impaired CD8^+^ T cell activation may hamper the development of protective cytotoxic T cell responses (41). Thus, the combination of HIV-1 infection induced T cell perturbations (42), and the observed increase in B_regs_ may further hinder control of HBV infection.

The costimulatory receptor CD86 is upregulated upon B cell activation and is a crucial costimulatory molecule for optimal T cell activation (43, 44). Viral infections lead to the expansion of CD21^–^T-bet^+^ B cells, which express high levels of CD86 and have similar phenotypes to AM and AtM B cells (45). T-bet^+^ B cells persist in chronic viral infections, such as HIV-1 and CHB, and are thought to be a source of antiviral immunoglobulin (9, 45) but are also associated with B cell exhaustion in the context of chronic antigen exposure (21, 46). In CHB, T-bet expression is increased in AtM B cells with higher expression frequencies of inflammatory homing markers CD11c and CXCR3 (9). While we did not measure T-bet, the combination of increased abundance of AtM B cells and elevated expression of CD86 on various B cell subsets within MWH with CHB may reflect the expansion of an activated, antiviral B cell population driven toward exhaustion by aberrant trafficking to inflamed sites such as the liver in CHB.

AtM B cells are present in circulation of healthy individuals (3%–5% total B cells) and may represent a short-lived lineage that can expand upon infection or vaccination (47, 48). However, during persistent antigen exposure, they can expand to over 50% and appear terminally differentiated and functionally inferior, with upregulation of exhausted-like inhibitory markers and less somatic hypermutation than their RM counterparts (21, 49, 50). Consistent with a negative functional consequence, we observed significantly increased AtM B cell frequencies in MWH with CHB compared with controllers. Of note, AtM B cell expansion has been reported in multiple long-term CHB monoinfection cohorts (9, 10, 51), whereas we see expansion only in HBV/HIV coinfection. Previous studies were conducted with participants many years after developing CHB while our samples were collected relatively shortly after acute infection (within 2 years, mean time 1.66 years). The discrepancy between our data and previous reports may reflect prolonged antigenic exposure in HBV monoinfection. Clinically, infection with HIV-1 prior to HBV exposure increases an individual’s risk of developing CHB 6-fold (16, 17) and within the MACS cohort, MWH not on active HBV-ART had a higher proportion of those with incident HBV develop CHB (17.5%) compared with MWoH (8.2%) (26). In addition, HIV-1 coinfection accelerates CHB-associated end-stage liver disease, cirrhosis, and HCC (52). Thus, AtM expansion in HIV-1 coinfection, as well as the other phenotypes we observed only in MWH, may reflect acceleration of a process that occurs over a longer period in CHB without HIV-1 coinfection. This hypothesis is supported by our results in MWH with low CD4^+^ counts showing significantly more pronounced immune perturbations in CHB.

A key feature of our B cell panel is the inclusion of HBsAg probes to identify ex vivo HBsAg-specific B cells longitudinally. The analysis of HBsAg-specific B cells also revealed a negative impact of HIV-1 on HBV, with limited HBsAg-specific B cell expansion in MWH. This failure of HBsAg-specific B cell expansion may restrict the anti-HBsAg response in coinfection. In support of this finding, clinical studies demonstrate that adults with HIV-1 mount a limited anti-HBsAg response to standard recombinant HBV vaccination compared with healthy individuals (53). In addition, HIV-1 infection leads to higher rates of reactivation of controlled HBV infection (18, 19), which may be partially attributable to the fact that people with HIV-1 are unable to sufficiently expand their HBsAg-specific B cell pool to maintain long-term HBV control. Although MWH failed to expand HBsAg-specific B cells, our cohort consisted of 31 participants who controlled their incident HBV infection. This suggests that in these individuals, additional immune populations, such as effector T cells, may be involved in HBV control. New evidence suggests TCF-1^+^CD127^+^PD-1^+^ precursor to exhaustion (Tpex) HBV-specific CD8^+^ T cells may contribute to control in the context of HIV/HBV coinfection (54).

In contrast, we found that in MWoH, HBsAg-specific B cells did expand, with significantly increased frequencies in controllers within 6 months to 2 years after acute infection. We did not see this B cell expansion during acute infection, when productive immune responses against HBV are first presumably being mounted, suggesting an overall delay in HBsAg-specific B cell induction and highlighting the potential importance of HBV-specific T cell responses during acute HBV control. A technical limitation to the HBsAg probe approach is that in samples with endogenous anti-HBsAg bound to HBsAg (acute and CHB samples), our ability to fully quantify HBsAg-specific B cells when antibody is produced was likely negatively affected. However, if the detection of HBsAg-specific B cell expansion in MWoH controllers were solely due to loss of HBsAg, we would also expect to see similar expansion in MWH controllers.

Unlike previous reports that characterize HBsAg-specific B cells in CHB (9, 10, 13–15), we focused our phenotypic analysis on intraparticipant expanded HBsAg-specific B cells to interrogate potential signatures of productive antigen-specific responses in HBV. Expanded HBsAg-specific B cells were predominantly in MWoH controllers at outcome and consisted mainly of class-switched (non-IgM expressing) IM and RM subsets. IM and RM antigen-specific B cells have been shown to expand upon control of SARS-CoV-2 infection (55) and are important for recall response to reinfection (56). In addition, HBsAg-specific B cells in CHB with treatment-associated HBsAg loss or seroconversion (functional cure) exhibit higher proportions of CD21^+^CD27^+^ RM B cells, suggesting this population is important for maintaining HBV control (14, 15). High-dimensional single-cell approaches like the method we employed enables a more granular assessment of B cell subsets. For example, despite their shared memory B cell annotation, expanded IM and RM subsets revealed heterogenous phenotypes, suggesting the definition of MBC may go beyond conventional CD21 and CD27 expression patterns. We observed a group of clusters with either fewer (#16 and #27; CXCR5^+^, CD86^–^, PD-1^–^, FcRL5^–^, CD11c^–^) or greater (#23 and #24; CXCR3^+^, CD86^+^, BTLA^+^, FcRL5^+^, CD11c^+^) activated signatures. Interestingly, these activated clusters appear phenotypically similar to AtM B cells based on the increased expression of FcRL5 and CD11c and downregulation of CXCR5. Therefore, our data suggest an activation and differentiation continuum within the HBsAg-specific B cell compartment, which may be required for productive and sustained HBV control in MWoH.

This study had multiple limitations due to the cohort studied. First, the findings described here were generated from an all-male cohort and may differ in women as immune responses are known to differ by sex. Second, since the participants came to semiannual visits, sampling at the peak of their acute infection was not always feasible, and the time between the acute infection and the sampling is not uniform. Third, all samples were obtained from peripheral blood draws, limiting our assessment to circulating B cells rather than the liver, the site of HBV infection. However, previous studies have identified phenotypes of circulating B cells to be present but enriched in the liver in HBV infection (9). Finally, the limited cell numbers available for each participant precluded subsequent in vitro testing to further interrogate functional consequences of the phenotypic signatures described by the ex vivo analysis.

Together, these results identify HIV-associated B cell alterations that elucidate potential mechanisms for previously identified negative effects of HIV-1 on HBV control. Multiple B cell subsets associated with dysregulation in antiviral responses were significantly upregulated in CHB in MWH (Figures 2 and 3) alongside failure to expand an antigen-specific B cell response. In contrast, global B cells in HBV monoinfection showed no differences by HBV outcome, and HBsAg-specific B cells expand during HBV control. These antigen-specific B cells consist mainly of IM and RM subsets with heterogenous activation phenotypes. Our observations support a role for B cells in HBV control of acute infection and warrant further functional examination to fully characterize HBsAg-specific B cells in acute and ongoing HBV control.

## Methods

### Sex as a biological variable.

All participants in this study were XY male and identified as men. This choice was guided by the access and availability of samples in the all-male MACS cohort; however, men demonstrate a higher incidence of CHB and HCC with poorer prognosis compared with women (57, 58).

### Study participants.

Male participants followed semiannually in the MACS (24) (now part of the MWCCS) (23) who had, or were at risk for, HIV-1 infection and had an incident HBV infection during follow-up were considered for inclusion. In the MACS, biological specimens including plasma and PBMCs are collected and stored at each study visit. Incident HBV was identified through retrospective serologic analysis, as previously described (25). Briefly, incident HBV infection was defined in participants who were negative for HBsAg and anti-HBc who seroconverted to anti-HBc positivity during follow-up. The estimated date of incident HBV infection was defined as the midpoint between the last negative and first positive anti-HBc or -HBsAg test result, whichever was positive first (25). The outcome of the incident HBV infection, HBV recovery (controller) or CHB, was defined based on serologic testing, as previously described (26). Briefly, CHB was defined by at least 2 positive HBsAg tests ≥ 6 months apart, and controller was defined as at least 1 visit negative for HBsAg and positive for anti-HBc at a visit within 18 months after the incident infection.

Men with incident HBV were included in this study if PBMCs were available prior to incident HBV infection (preinfection, –0.5 to 0 years from estimated incident infection), at the first visit positive for anti-HBc or HBsAg (acute infection, 0 to 0.5 years), and/or 0.5–2 years after the estimated date of incident infection (early outcome, 0.5 to 1.5 years; late outcome, 1.5 to 2 years) (Figure 1A and Tables 1 and 2). We matched all men with known CHB who had PBMCs available at these time points to men with known HBV recovery (controllers) at a minimum 1:1 (CHB to controller, 4 groups), but where possible, we matched 1:2 (10 groups), 1:3 (8 groups), or 1:5 (1 group) based on age, HIV-1 status, CD4^+^ T cell count (for MWH), and storage time of samples in the repository. We included 76 men of whom 54 were HBV controllers and 22 had CHB. Of the 76 men included, 44 were living with HIV-1 and 32 were not for all time points assessed. For most participants included in the study, seropositivity for HBsAg, anti-HBc, anti-HBsAg, and HBV DNA levels are available, as well as ART for those participants receiving HIV-1 treatment (*N* = 8) (Tables 1 and 2). HBV DNA was measured only at the acute HBV time point. All participants on treatment remained so throughout the entire time course assessed here. HIV viral load and CD4^+^ counts were also measured at all time points and did not significantly differ by HBV infection status. Not all participants had samples available for all 4 time points (21 participants lacked 1 of 2 outcome samples, 9 participants lacked samples from both outcome time points, and 4 participants did not have a preinfection sample available). A total of 262 samples were included for processing and staining.

### Sample processing and flow cytometry staining.

PBMCs were cryopreserved in FBS + 10% DMSO and stored in liquid nitrogen until use in this study. PBMC processing and flow staining were performed using the pipeline previously described (59). Briefly, cryopreserved PBMCs were thawed in 37°C prewarmed RPMI + 10% FBS (R10) using the Thawsome adaptor (Medax International, Inc.) (60). Cells were washed once in 5 mL R10, then centrifuged at 860*g* for 3 minutes and transferred to V-bottom, 96-well plates for immediate B cell panel staining.

All flow cytometry antibodies and staining reagents used for this study are in Supplemental Table 2 and are further described in detail in Cascino et al. 2020 (22). The panel was designed to define comprehensive B cell subsets (Supplemental Figure 1). For the antigen-specific B cell probe, we obtained recombinant small HBsAg genotype C, manually conjugated to 1 of 2 fluorochromes, Dylight 550 or Dylight 650, from Gilead Sciences (22, 61).

Antibody staining cocktail containing all but the antigen-specific B cell probes was prepared in advance and stored at 4°C before staining the next day. Immediately prior to B cell staining, 0.2 μg/50 μL reaction of each antigen-specific B cell probe was added and centrifuged at 15,000*g* for 5 minutes to remove dye aggregates. PBMCs were washed twice in 200 μL phosphate-buffered saline (PBS) prior to staining with Fixable Live/Dead Aqua (Thermo Fisher Scientific) for 20 minutes at 4°C. Samples were then washed twice in 200 μL PBS and stained with 50 μL staining cocktail for 30 minutes at 4°C in the dark. After incubation, samples were washed twice with 200 μL staining buffer (PBS + 0.5% bovine serum albumin) and subsequently fixed for 20 minutes with 2% paraformaldehyde (PFA) at 4°C. After fixation, cells were washed once with staining buffer, resuspended in 250 μL 0.5% PFA, and stored at 4°C in the dark until acquisition the next day on a BD FACSymphony A5 cytometer using FACSDiva software (version 9.3.1). The flow cytometers were standardized to ensure consistent and reproducible performance according to Perfetto et al. (62, 63).

The total 262 samples were processed in 4 batches. Matched participants were randomized to 1 of the 4 batches, and all samples for those participants were included in the same batch to mitigate potential issues with batch effects. PBMCs from the same blood draw from an unexposed healthy individual were measured in all experiments as a technical control to assess reproducibility and for data normalization (59).

### Flow cytometry data pre-processing.

Flow cytometry data pre-processing is critical to remove low-quality data and prevent artificial data output. We used the R-implemented (R version 4.0.0) algorithm FlowAI (version 1.18.5) (59) for automated removal of irregular events and outliers, which can arise from fluctuations in flow cytometer performance. In 222 out of 262 samples, FlowAI detected minor irregular events (<10%), which were removed automatically. For the remaining 40 samples with more than 10% irregular events, we manually inspected the raw data to prevent excessive data loss (64). Of these samples, 20 required manual removal of irregular events.

Subsequently, correction for spectral overlap (compensation) was performed in FlowJo 10.1.7 (BD Biosciences) using single-stained beads. Persisting compensation inaccuracies were corrected manually.

We performed preliminary gating of viable CD19^+^CD20^+^ B cells with FlowJo 10.9 and exported a new set of FCS files for subsequent analysis of immune cell characteristics. The precision of flow cytometric analysis depends on Poisson statistics and thus requires sufficient cell counts. To this end, 4 samples were excluded from analysis due to limited B cell counts (<5,000 B cells). Also, 14 additional samples were removed from analysis to avoid duplication of samples in each of the 4 longitudinal groups (preinfection, acute, early outcome, late outcome) from a single participant. A total of 244 samples were included for analysis moving forward.

### Manual flow cytometry analysis.

Manual gating was performed using FlowJo 10.9. Markers were divided in 2 groups based on their purpose to define immune cell subsets (lineage markers) or functional markers/characteristics (Supplemental Table 2). The manual gating strategy can be found in Supplemental Figure 1 and Cascino et al. (22).

Three parameters were extracted for subsequent analysis: frequency of immune cell populations, frequency of cells expressing functional markers, and geometric mean fluorescence intensity (MFI).

### Unsupervised data analysis.

Based on the technical control samples, we observed batch-to-batch variation between the 4 experiments. Our manual gating analysis accommodates for batch variation by applying cutoff gates on a per-experiment basis. However, nonbiological interassay data variation will negatively influence the accuracy of unsupervised data analysis (27, 59). Therefore, we normalized the flow cytometry raw data using CytoNorm (65). To this end, we concatenated all files per experiment to train the CytoNorm model, which was applied to the aggregated files as described (66). The normalized flow cytometry data were used for subsequent unsupervised data analysis with concatenated FCS files. Samples were identifiable by sample-specific keywords. R (version 4.2.1) programming language with RStudio (2023.06.0 Build 421) interface was used for unsupervised data analysis. Raw data (FCS files) containing concatenated B cells were imported using the flowCore (version 2.10.0) package. Data were transformed using the estimateLogicle function from the flowCore package.

For dimensionality reduction algorithm UMAP (version 0.2.10.0) (67) and FlowSOM clustering analysis, we combined all HBsAg-specific B cells (*N* = 5,321) with 1,000 randomly selected B cells per sample (total 244,000 B cells from 244 samples; 109,000 B cells from MWH, 135,000 B cells from MWoH) to generate UMAPs and define cluster abundances for total and antigen-specific B cells from all time points. HBsAg-specific B cells were defined based on costaining of the 2 B cell probes. A cutoff of 1.8 and 1.9 was used for logicle-transformed signal from Dylight 650– and Dylight 550–conjugated probes, respectively (Figure 4, C and D). For UMAP, default settings were used, and all markers except the HBsAg-specific B cell probes and viability/dump markers were used. To visualize expression levels on the UMAP, logicle-transformed fluorescence intensity values were normalized based on 1% and 99% percentile. Figure 1B is based only on preinfection total B cell composition between MWoH and MWH. Here, UMAP was visualized based on 800 and 1,000 randomly selected B cells per MWH (*N* = 40) and MWoH (*N* = 32) to obtain equal numbers of B cells between HIV-1 infection status (*n* = 32,000 cells per group). In Figure 1E, Figure 2A, and Supplemental Figure 3B, UMAP algorithm based on 16 non-normalized manually gated B cell subset frequencies were used to define unique immune compositions between MWH and MWoH (Figure 1E and Supplemental Figure 3B) and controller and CHB (Figure 2A) where each dot represents 1 sample.

For visualization of HBsAg-specific B cells in either UMAP or biaxial raw data, 1 participant was removed because of large expansion of HBsAg-specific B cells, which affects the visualization of rare HBsAg-specific B cells from other samples. The HBsAg-specific B cells from this participant were included for statistical analysis.

Clustering of B cells from all 244 samples (*N* = 17,432,605) was performed using FlowSOM (version 2.4.0). FlowSOM parameters included a cluster grid of 20 × 20, and in a subsequent step 40 metaclusters were defined. Fluorescence signals from all markers (*N* =21) except for viability dye and the 2 antigen-specific B cell probes were used to compute FlowSOM clustering. Manual gating designation was extracted from FlowJo workspace using the GetFlowJoLabels function from the FlowSOM package.

The heatmap showing MFI for each marker per cluster (Supplemental Figure 4B) was created with the pheatmap package (version 1.0.12), and input data were normalized using 1% and 99% percentile normalization. The ggplot2 (version 3.4.4) package was used for data visualization. Overlay histograms depicting expression of markers per cluster was performed using the ggridges package (version 0.5.5). To this end, 10,000 B cells were randomly selected across all samples to visualize expression on total B cells.

### Statistics.

Statistical comparisons were performed in GraphPad Prism 10 or R. Comparisons between conditions were performed using nonparametric tests with or without correction for multiple testing as indicated in figure legends. A *P* value less than 0.05 was considered significant, except for analysis in Figure 5B, where we visualized FlowSOM clusters, which had at least 1 significant difference with a *P* < 0.01 to highlight the differences that remained statistically significant using a more conservative type 1 error threshold.

For the exploratory analyses in Figure 1G; Figure 2, B–D; Figure 3, A and B; and Figure 5A, we tested the difference of 40–370 unique immune characteristics between CHB and HBV controllers at 4 time points (Supplemental Table 1). Thus, our sample size is not sufficiently large for multiple-testing correction with such comprehensive lists of distinct immune characteristics. Therefore, we focused our analysis on specific immune characteristics as follows. For Figure 1G, we show all phenotypic immune characteristics with a *P* < 0.05 by Wilcoxon’s signed-rank test (BTLA, FcRL5, and CD22 demonstrated no significant differences). For the remaining figures, immune characteristics we selected to highlight in the figures were only those characteristics that significantly differed in consecutive time points (Figure 2, B and C; Figure 3, A and B; and Figure 5A) and/or between multiple immune characteristics (manually gated frequency and MFI; Figure 2D). Finally, for the analysis in Supplemental Figure 2B, we first assessed the differences between the 2 controller groups and CHB at the acute time point using Kruskal-Wallis rank test. Those significant characteristics were then compared between the acute groups by Wilcoxon’s signed-rank test in Supplemental Figure 2C, which primarily revealed CD86 expression to be significantly different. Thus, we focused our analysis in Supplemental Figure 2D on CD86 expression in various B cell subsets to visualize significance of CD86 expression between all 3 acute groups (controller 1, controller 2, and chronic) as determined by Wilcoxon’s signed-rank test.

In Figure 5, frequency of HBsAg-specific B cells within the 40 B cell clusters was calculated. Wilcoxon’s signed-rank test without correction for multiple testing was used to identify significant differences in HBsAg-specific B cell abundance within FlowSOM clusters between time points and per HIV infection status and HBV outcome (Figure 5A and Supplemental Figure 5F). For multiple clusters (#2, #17, #19, #22, #32, #34, #35, #36, #38, and #39), differences could not be assessed due to insufficient HBsAg-specific B cells between the 2 groups in at least 1 sampling time point.

We identified 12 FlowSOM clusters in Figure 5A with at least 1 significant difference (*P* < 0.01). Of these clusters, we selected to further analyze 10 clusters of interest in Figure 5B under the following criteria: the 2 clusters significantly upregulated for MWH (clusters 10 and 15) and only the 8 clusters significantly upregulated at 2 or more time point comparisons #4, #9, #16, #23, #24, #27, #28, and #33). In Figure 5B we then performed Wilcoxon’s signed-rank test with Bonferroni’s correction for multiple testing to determine statistically significant differences in longitudinal HBsAg-specific B cell abundances in our clusters of interest. Of note, the remaining clusters from Figure 5A (clusters 13 and 18) did not show any significant differences between time points after Bonferroni’s correcting for multiple testing.

Finally, to incorporate all available longitudinal data in comparisons of expression levels across multiple time points, we fit GEE models using SAS GENMOD procedure, which accounts for the resulting correlated data structure (Figure 2E and Figure 3E). In these GEE analyses, we modeled the natural-log-transformed immune expression data as the dependent variable and chose to account for the correlated data using an autoregression correlation structure after examining model fit results.

### Study approval.

All participants provided written informed consent for this study. This study was approved by the Institutional Review Board at Johns Hopkins University.

### Data availability.

Data values for all graphs are provided in the Supporting Data Values file.

## Author contributions

KC and TL (co–first authors) contributed equally to designing and conducting experiments, acquiring and analyzing data, and preparing figures for publication. KC had primary responsibility for planning, scheduling experiments, and drafting the manuscript and is therefore assigned as lead co–first author. ECS contributed to data interpretation. KES contributed to flow cytometry panel design. SMW, MDW, and RBM contributed to sample acquisition and manuscript review. MR provided facilities and contributed to manuscript review. JRB contributed to data interpretation and manuscript review. ALC and CLT conceived and supervised the study and contributed to data analysis, data interpretation, and manuscript review.

## Conflict of interest

The authors have declared that no conflict of interest exists.

## Funding support

This work is the result of NIH funding, in whole or in part, and is subject to the NIH Public Access Policy. Through acceptance of this federal funding, the NIH has been given a right to make the work publicly available in PubMed Central.

- Vaccine Research Center, an intramural Division of NIAID, NIH.
- Foundation for the National Institutes of Health through the Collaboration for AIDS Vaccine Development of the Bill & Melinda Gates Foundation grant OPP1147555 to TL and MR.
- NIAID grant R01AI116269 to CLT and ALC.
- Grant U19 AI188551.
- MWCCS by the National Heart, Lung, and Blood Institute (NHLBI), with additional cofunding from the Eunice Kennedy Shriver National Institute of Child Health & Human Development (NICHD), National Institute on Aging (NIA), National Institute of Dental & Craniofacial Research (NIDCR), National Institute of Allergy and Infectious Diseases (NIAID), National Institute of Neurological Disorders and Stroke (NINDS), National Institute of Mental Health (NIMH), National Institute on Drug Abuse (NIDA), National Institute of Nursing Research (NINR), National Cancer Institute (NCI), National Institute on Alcohol Abuse and Alcoholism (NIAAA), National Institute on Deafness and Other Communication Disorders (NIDCD), National Institute of Diabetes and Digestive and Kidney Diseases (NIDDK), and National Institute on Minority Health and Health Disparities (NIMHD), and in coordination and alignment with the research priorities of the National Institutes of Health, Office of AIDS Research (OAR).
- MWCCS data collection by UL1-TR000004 (UCSF CTSA), UL1-TR003098 (JHU ICTR), UL1-TR001881 (UCLA CTSI), P30-AI-050409 (Atlanta CFAR), P30-AI-073961 (Miami CFAR), P30-AI-050410 (UNC CFAR), P30-AI-027767 (UAB CFAR), P30-AI-124414 (ERC-CFAR), P30-MH-116867 (Miami CHARM), UL1-TR001409 (DC CTSA), KL2-TR001432 (DC CTSA), and TL1-TR001431 (DC CTSA).

## Supplementary Material

Supplemental data

ICMJE disclosure forms

Supplemental table 1

Supporting data values

## Figures and Tables

**Table 2:**
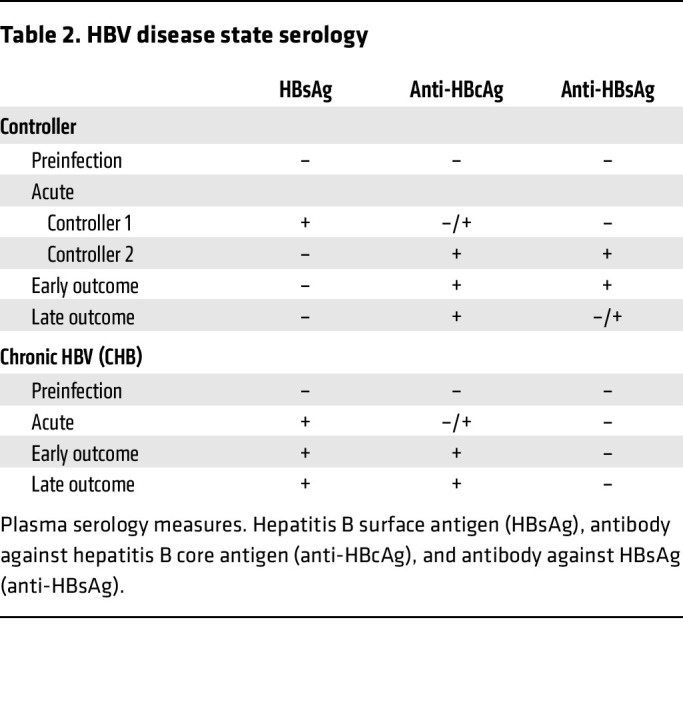
HBV disease state serology

**Table 1:**
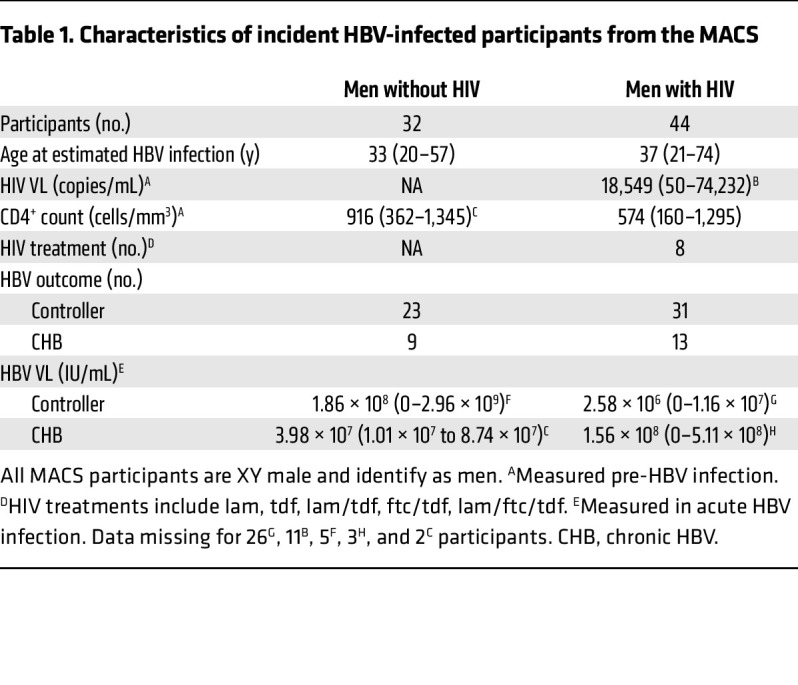
Characteristics of incident HBV-infected participants from the MACS

## Version 1 04/07/2026

In-Press Preview

## Version 2 06/01/2026

Electronic publication
