# Supplementary material for: HIV causes global B cell dysregulation and restricts HBV-specific B cell development in an incident HBV cohort
Source: J Clin Invest. 2026 Apr 7;136(11):e203138. doi: 10.1172/JCI203138 (PMC13221222; doi:10.1172/JCI203138)
Supplement: Supplemental data [file jci-136-203138-s218.pdf]

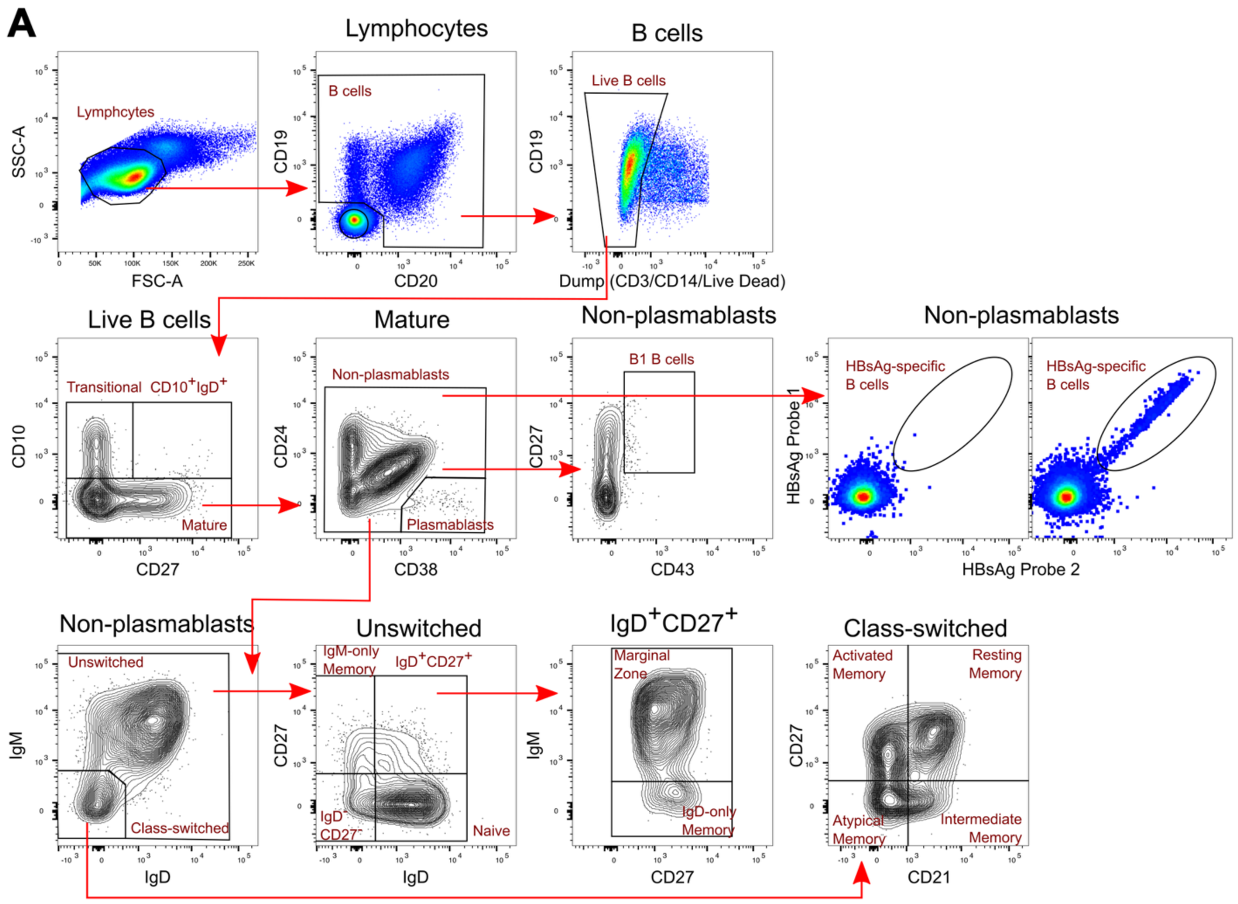

Supplemental Figure 1
(A) Representative flow cytometry plots from a MACS participant (MWH, HBV controller) at the acute time point show gating of all B cell subsets assessed by the B cell panel. An additional flow cytometry plot for HBsAg-specific B cells from a MWoH, HBV controller at the late outcome time point shows example of high frequency HBsAg-specific B cells.

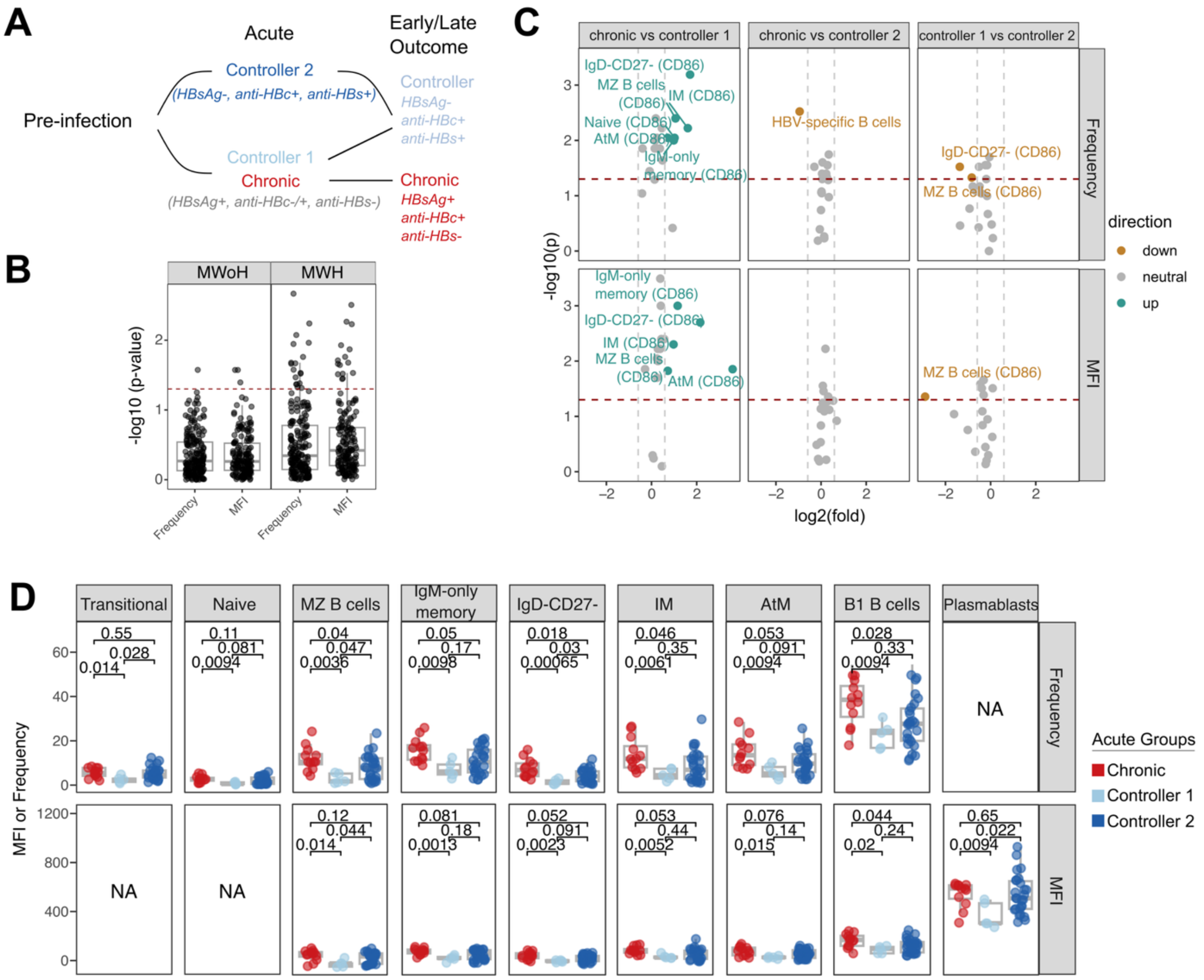

Supplemental Figure 2

(A) Schematic representation of three groups (controller 1, light blue; controller 2, dark blue; and chronic, red) present at acute time point. Groups are defined by serologic analysis and current or future known HBV infection outcome. **(B)** Kruskal-Wallis rank test of immune characteristics between controller 1 or chronic and controller 2. Immune characteristics consist of manually gated immune cell frequencies (Frequency) or receptor expression levels (Geometric mean; MFI). Plot shows results for MWoH (left) and MWH (right) individuals. Each dot represents one immune characteristic. **(C)** Volcano plots show comparison of all manual gated frequencies (top row) and MFI (bottom row) for MWH at acute time point compared between controller 1 vs chronic (column 1), controller 2 vs chronic (column 2), and controller 1 vs controller 2 (column 3). Each dot represents a characteristic that was determined as significantly different by Kruskal-Wallis from B. Teal dots represent significantly increased characteristics and orange dots are significantly decreased characteristics. Y-axis depicts -log10 transformed unadjusted p-values based on Wilcoxon signed-rank test. **(D)** Comparison of CD86 on B cell subsets for which controller 1 vs chronic comparison demonstrated significantly different values. (controller 1, light blue; controller 2, dark blue; and chronic, red). All samples are assessed from MWH using Wilcoxon signed-rank test

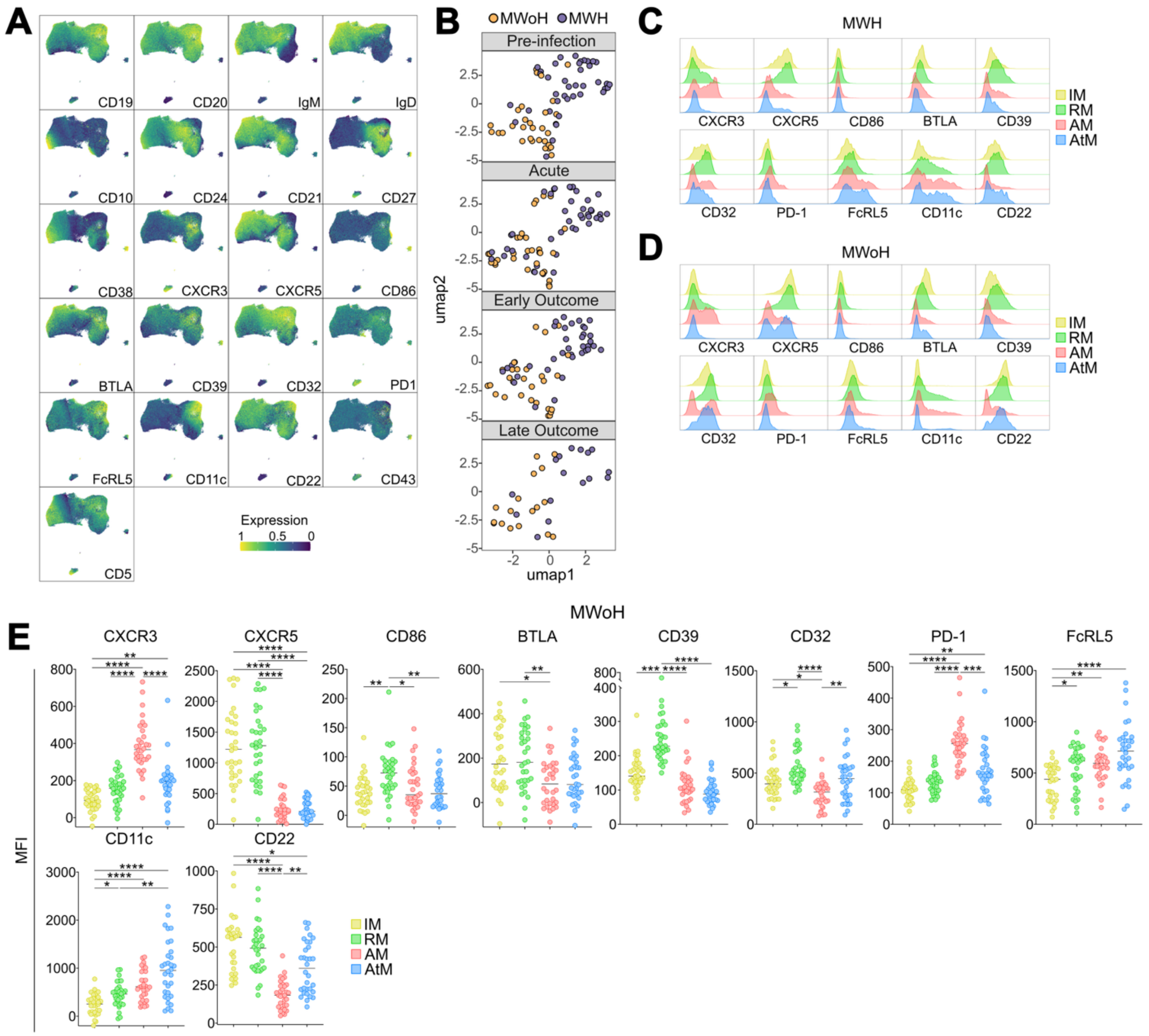

Supplemental Figure 3

(A) MFI expression for all markers in the panel overlaid on UMAP of total B cells. **(B)** UMAP projection based on abundance of manually gated B cell subsets shown in Figure 1, C and D at all time points for MWoH (yellow) and MWH (purple). Each dot represents one participant at a given time point. **(C-D)** Representative MWH **(C)** and MWoH **(D)** from the pre-HBV infection time point examined for expression levels of phenotypic markers on the four MBC subsets defined based on CD21/CD27 expression profile: IM ($CD21^{+}CD27^{-}$; yellow), RM ($CD21^{+}CD27^{+}$; green), AM ($CD21^{-}CD27^{+}$; red) and AtM ($CD21^{-}CD27^{-}$; blue). **(E)** MFI of phenotypic markers on B cell memory subsets from all MWoH pre-HBV infection samples. Data compared using Kruskal-Wallis test corrected for multiple comparisons and each dot represents a single measurement from each participant assessed at the pre-HBV infection time point. Bars represent median values. *, $P < 0.05$; **, $P < 0.01$; ***, $P < 0.001$; ****, $P < 0.0001$.

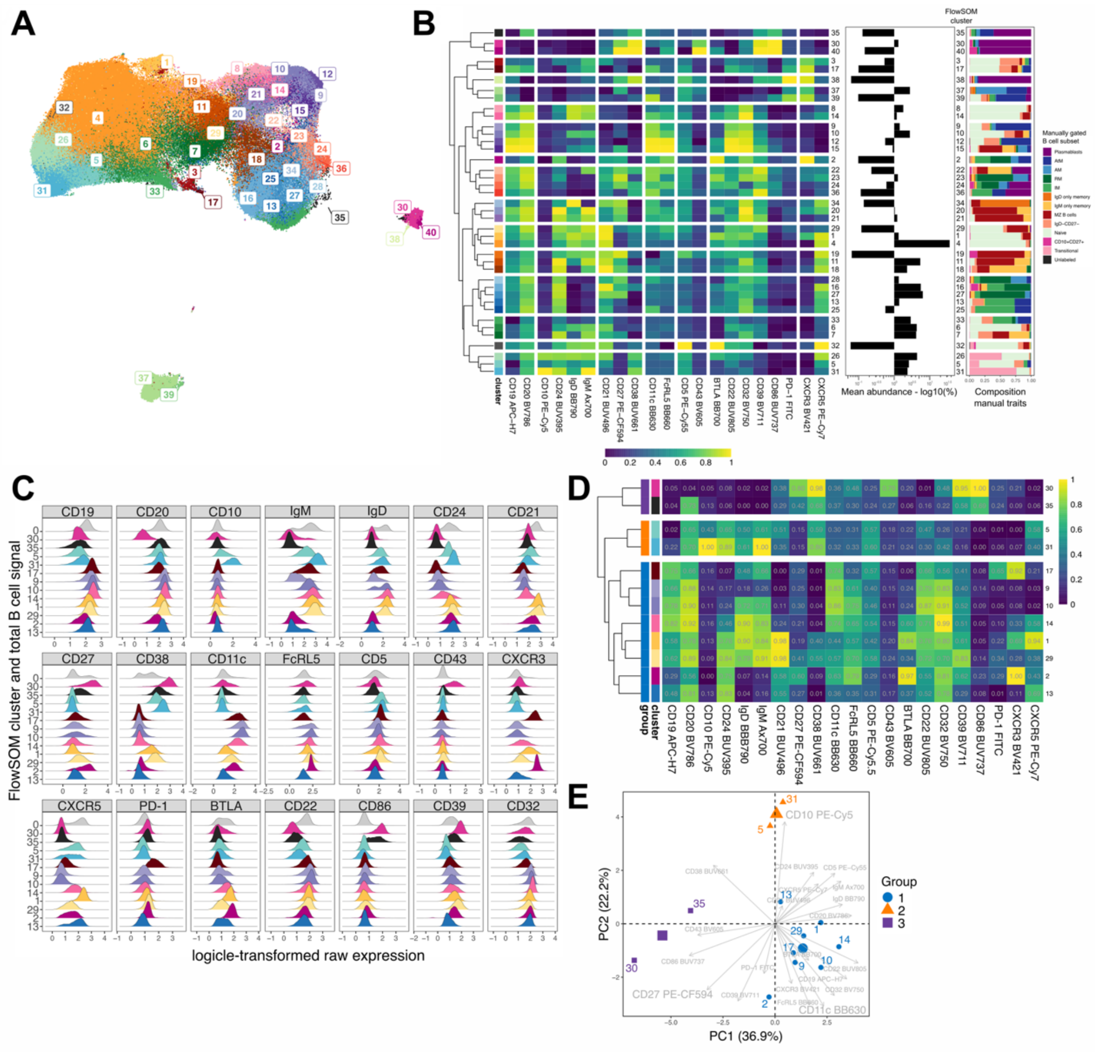

Supplemental Figure 4

(A) FlowSOM clustering based on all CD19⁺ B cells (n = 17,432,605) from all 244 samples with a total of 40 clusters identified. Subsetted (1000 CD19⁺ B cells per sample and all 5321 HBsAg-specific B cells) and concatenated flow cytometry data is depicted as UMAP projection and colored based on FlowSOM cluster annotation. **(B)** Heatmap shows relative expression (99th percentile normalization) of all markers for each of the 40 FlowSOM clusters. Heatmap rows (FlowSOM clusters) are clustered based on similar marker expression profile based on Euclidean hierarchical clustering and divided into 16 clusters. Black bar graph depicts mean abundance and colored bar graph depicts composition of manual gated subsets per FlowSOM cluster. **(C)** Histogram plots visualize marker expression (MFI) for 12 FlowSOM clusters identified to be upregulated in controllers compared to CHB (Figure 3A). Expression profile from total B cells as a reference is shown as grey histograms. **(D)** Heatmap depicts relative expression of all markers for the 12 significant FlowSOM clusters shown in C. Euclidean hierarchical clustering was used to define 3 groups with unique expression profiles. Numbers represent MFI expression values. **(E)** Principal component analysis (PCA) for the three FlowSOM cluster groups defined in D based on all phenotypic markers highlights distinctive markers between each group identified in grey.

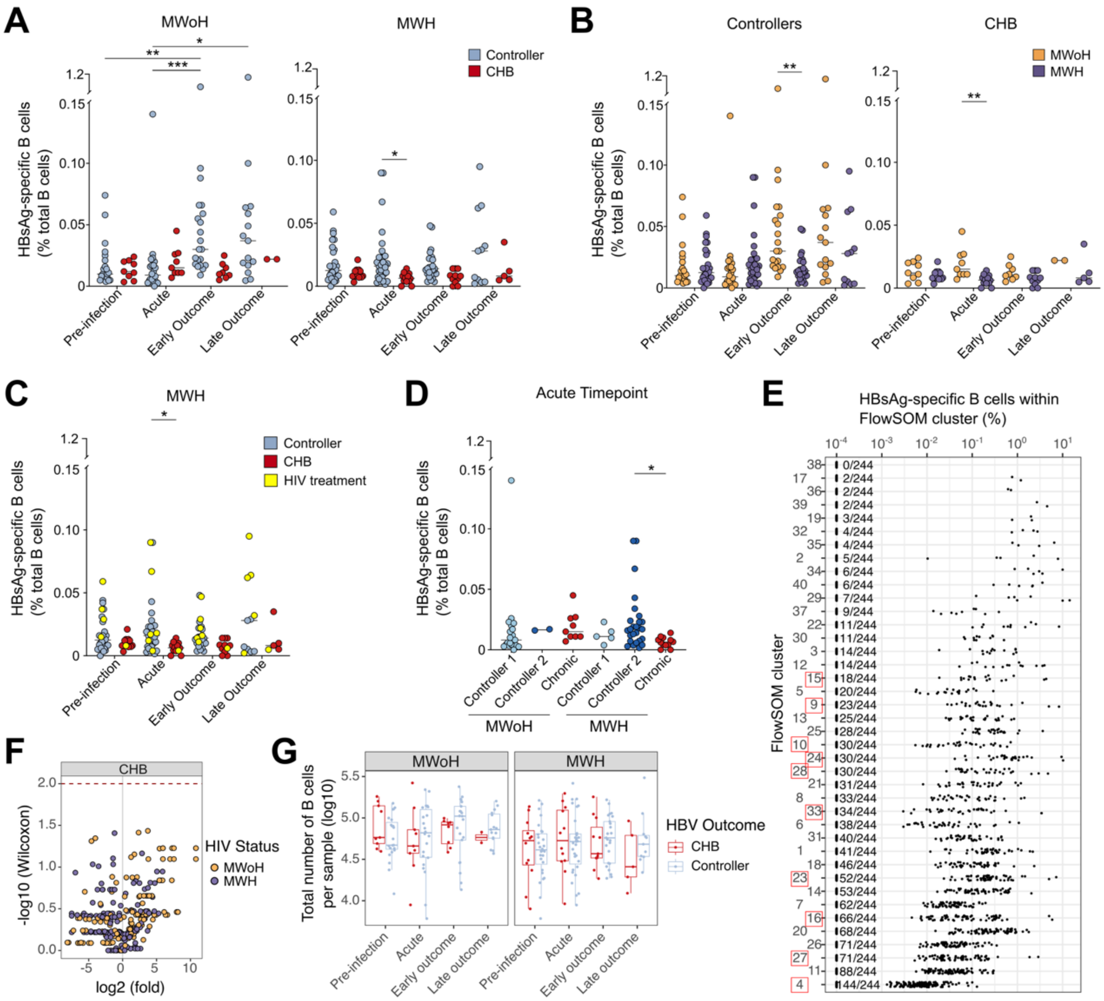

Supplemental Figure 5

(A-B) HBsAg-specific B cell frequencies of total B cells separated by **(A)** HIV-1 infection status (MWoH and MWH) and compares controller to CHB or **(B)** HBV outcome (controllers and CHB) and compares MWoH to MWH. **(C)** Frequencies of HBsAg-specific B cells only in MWH. Yellow dots represent participants on HIV-1 treatment (N = 8). **(D)** Frequencies of HBsAg-specific B cells at acute time point stratified by acute group (controller 1, light blue; controller 2, dark blue; chronic, red) and HIV-1 infection status. Definition of controller groups is shown in Supplemental Figure 2. **(E)** Percent of HBsAg-specific B cells within each of 40 FlowSOM clusters. The number of samples out of 244 for which HBsAg-specific B cells are detected for a given FlowSOM cluster is also shown. Red boxes show 10 clusters examined in Figure 5. **(F)** Log2 fold-change of HBsAg-specific B cells by FlowSOM cluster in CHB calculated by Wilcoxon analysis are plotted by volcano plot. Y-axis represents -log10 transformed unadjusted p-values and dotted line visualizes significance threshold ($p < 0.05$). Analysis analogous to Figure 5A but all comparisons combined in one volcano plot. **(G)** Log10 total number of B cells acquired per sample is plotted for each time point stratified by HIV-1 infection status and HBV outcome. Each dot represents one sample at a given time point. **(A-D)** Data compared using Kruskal-Wallis test adjusted for multiple comparisons. Each dot represents a single sample at a given time point. *, $P < 0.05$; **, $P < 0.01$; ***, $P < 0.001$.

Supplemental Table 2. B cell panel antibodies

	Marker	Fluorochrome	Vendor	Clone	Cat #
Lineage Markers	CD19	APC H7	BD Biosciences	H1B19	560727
	CD20	BV786	BD Biosciences	TH7	743611
	CD10	PE Cy5	BD Biosciences	HI10a	555376
	CD38	BUV661	BD Biosciences	HIT2	565070
	CD24	BUV395	BD Biosciences	ML5	566221
	IgM	AF700	Biolegend	MHM88	314537
	IgD	BB790	BD Biosciences	IA6-2	624296
	CD27	PE CF594	BD Biosciences	M-T271	562297
	CD21	BUV496	BD Biosciences	B-ly4	624283
Functional Markers	PD-1	FITC	Biolegend	EH12.2H7	329904
	CD11c	BB630	BD Biosciences	BU15	624294
	FcRL5 (CD307e)	BB660	BD Biosciences	509F6	624295
	CD22	BUV805	BD Biosciences	HIB22	742009
	CD32 (FcγRII)	BV750	BD Biosciences	3D3	747110
	BTLA	BB700	BD Biosciences	J168-540	746166
	CD43	BV605	BD Biosciences	1G10	563378
	CD39	BV711	BD Biosciences	Tu66	563680
	CD86	BUV737	BD Biosciences	FUN1	612785
	CD5	PE Cy5.5	Thermo	CD5-5D7	MHCD0518
	CXCR3	BV421	Biolegend	G025H7	353715
	CXCR5	PE Cy7	Biolegend	J252D4	356923
HBsAg Probes	HBsAg	Dylight 550	Gilead	Genotype C	NA
	HBsAg	Dylight 650	Gilead	Genotype C	NA
Dump	CD3	BV510	BD Biosciences	UCHT1	563109
	CD14	BV510	BD Biosciences	MφP9	563079
